# Renal Phosphate Reabsorption in Humans Depends on at Least Three Distinct Transporters Unlike in Mice

**DOI:** 10.1111/apha.70271

**Published:** 2026-07-23

**Authors:** Ashley L. Fernandes, Laurine Lang, Jürgen Klar, Anja Giese, Alexander Ehrmann, Rupert Busch, Hannah Auwerx, Christoph Daniel, Nati Hernando, Isabel Rubio‐Aliaga, Carsten A. Wagner

**Affiliations:** ^1^ Institute of Physiology University of Zurich Zurich Switzerland; ^2^ Cardiovascular, Renal and Immunology Research Bayer AG Wuppertal Germany; ^3^ Drug Discovery Sciences Bayer AG Berlin Germany; ^4^ Drug Discovery Sciences Bayer AG Wuppertal Germany; ^5^ Department of Nephropathology Friedrich‐Alexander Universität (FAU) Erlangen‐Nürnberg Erlangen Germany

**Keywords:** human renal brush border membrane vesicles, renal phosphate cotransporter, SLC34A1, SLC34A3

## Abstract

**Aim:**

Kidney excretion of phosphate is the gatekeeper of systemic phosphate homeostasis as evident from inborn and acquired diseases. Renal phosphate transporters are a promising target for phosphate‐lowering drugs, but molecular details of human kidney phosphate handling are largely unknown. Here, we aim to understand the dependency of renal phosphate transport on species, age, and sex.

**Methods:**

We used a combination of transporter‐specific inhibitors and radioactive flux measurements in isolated BBM vesicles prepared from human and murine kidneys. We included human female and male neonates (0–11 months) and adults (51–63 years) and age‐matched mouse kidneys. Immunoblotting and immunofluorescence detected transport protein expression, and transcript expression was analyzed in publicly available data.

**Findings:**

The flux experiments revealed that in human kidneys SLC34A1, SLC34A2/3 and other non‐SLC34 transporter are active and expressed with age‐ and sex‐dependent differences. In mice about 80% of renal phosphate handling depends on Slc34a1, but in humans SLC34A1 contribution is 60% in neonates and only 40% in adults. SLC34A3 contribution accounts for 20% in human neonates and 40% in human adults but is almost negligible in mice. Non SLC34 sodium‐dependent phosphate transport was around 20% in all groups. SCRNA‐seq data and immunoblotting analysis revealed differences in sodium cotransporters between species that supported the activity measurements.

**Conclusion:**

Our data provide the first direct measurement of sodium‐phosphate cotransporter activities in human kidney and show profound differences between species. These results are critical when developing novel drugs to modulate renal phosphate reabsorption.

## 1 Introduction

Renal phosphate handling is critical for the control and regulation of systemic homeostasis of inorganic phosphate, which is relevant for the pathophysiology of cardiovascular and chronic kidney disease [1]. Phosphate is freely filtered by kidneys and reabsorbed mostly at the level of the proximal tubule. Reabsorption is mediated by sodium‐dependent phosphate cotransporters located in the brush border membrane (BBM), taking up phosphate from primary urine. The mechanisms by which phosphate is released from proximal tubules across the basolateral membrane are incompletely understood. Based on human transporters cloning experiments and animal studies, at least three distinct sodium‐dependent phosphate cotransporters have been localized to the proximal tubule BBM; NaPi‐IIa (Npt2a, *SLC34A1*) and NaPi‐IIc (Npt2c, *SLC34A3*) from the SLC34 family and Pit‐2 (*SLC20A2*) from the SLC20 family [2, 3]. These cotransporters differ in their transport mechanisms and hormonal regulation. NaPi‐IIa and Pit‐2 are electrogenic, while NaPi‐IIc is electroneutral. NaPi‐IIa and NaPi‐IIc prefer HPO_4_^2−^ as substrate, and PiT‐2 H_2_PO_4_^−^. NaPi‐IIa transfers 3 sodium ions per transport cycle and NaPi‐IIc and Pit‐2 transport 2 sodium ions [3]. All three cotransporters respond to changes in dietary phosphate intake, parathyroid hormone (PTH) and fibroblast growth factor 23 (FGF23), albeit with different time constants.

The relative contribution of these cotransporters to overall renal phosphate handling has been established based on knockout mouse models. Deletion of *Slc34a1* (NaPi‐IIa) reduced renal phosphate transport and reabsorption by approximately 80% [4, 5]. Likewise, pharmacological studies in rodents using an inhibitor specific to NaPi‐IIa caused profound phosphaturia [6, 7]. In contrast, several animal models with genetic ablation of *Slc34a3* (NaPi‐IIc) did not show any altered renal Pi handling [8, 9, 10]. Of note, the peak of protein expression levels of NaPi‐IIc in mouse kidney are around weaning and protein abundance in adult mouse kidney is very low [11]. NaPi‐IIb (*Slc34a2*) expression in mouse kidney appears to be restricted to the loop of Henle [12]. Genetic ablation of *Slc20a2* (Pit‐2) from rodent kidneys has not been reported to date. Thus, studies in rodents collectively suggest that NaPi‐IIa is the predominant renal Na^+^‐phosphate cotransporter in the proximal tubule accounting for most of renal Pi reabsorption.

In humans, biallelic loss‐of‐function variants in *SLC34A1* cause renal phosphate losses with subsequent hypophosphatemia, elevated calcitriol levels, hypercalcemia and hypercalciuria and nephrocalcinosis and ‐lithiasis [13, 14]. Symptoms start in utero and seem to persist at least into adolescence and early adulthood [15]. Similarly, biallelic loss‐of‐function variants in *SLC34A3* cause comparable clinical manifestations that may also include rickets [16, 17]. The symptoms in these patients clearly persist into adulthood [15, 16, 17]. Monoallelic loss‐of function variants in *SLC20A2* (Pit‐2) cause familial basal ganglia calcification, but a systemic or renal phenotype has not been reported in these patients [18]. Thus, human genetics suggest that both NaPi‐IIa and NaPi‐IIc play important roles in renal phosphate handling and that they cannot fully compensate each other's absence. Also, there might be an age‐dependent switch from NaPi‐IIa to NaPi‐IIc activity, as suggested by the partial amelioration of symptoms in patients with *SLC34A1* variants.

Age‐dependent changes in renal phosphate handling have been demonstrated in several cohorts and show a progressive reduction in TmP/GFR, a measure of the capacity of the proximal tubule to reabsorb phosphate, from infants to adults [19, 20]. However, in women around the age of 35–50 years, TmP/GFR rises again, while it continues to fall in men, suggesting also important age‐dependent sex differences [19].

Based on the observed apparent differences between mice and humans, we sought to resolve these discrepancies by testing the activity of different sodium‐dependent phosphate cotransporters in mouse and human kidneys using the BBM vesicle (BBMV) filtration technique and SLC34 inhibitors. Our data are the first to directly examine human renal phosphate transporters in their native environment. We compared kidneys from adult human female and male donors (50–64 years old) with age‐matched adult mice, and from neonate humans (0–11 months) with kidney maturation‐matched mice (weaned). Our results reveal species, age, and sex differences that have implications for our understanding of renal handling of phosphate and associated disorders as well as for the development of drugs targeting these cotransporters in conditions associated with elevated phosphate levels.

## 2 Methods

### 2.1 Human Kidneys

Twenty one non‐transplantable human kidneys from adult and 13 from neonate subjects were acquired from the International Institution for the Advancement of Medicine (IIAM, USA, https://iiam.org/) and stored at −80°C. Kidneys from adult donors with recent SARS‐CoV‐2 infections, HIV and Hepatitis B/C infections were not acquired. Next, the following parameters were used to determine whether all adult kidneys were suitable for analysis: estimated glomerular filtration rate (eGFR), calculated from serum creatinine values obtained during hospitalization and up to kidney donation, using the CKD‐EPI Creatinine Equation (2021) considering body area [21]. Initial and final eGFR values, as well as the change over this interval, were assessed. In addition, the protein expression levels of COL1A1, KIM‐1, and NGAL were quantified by densitometry analysis to evaluate renal fibrosis and injury (Figure S1). Eleven adult kidneys met the inclusion criteria and were used for this study (Table S1). For neonates, we excluded subjects with genetic conditions that could affect kidney (e.g., trisomy). Four groups were analyzed: adult males and females, 51–63 years old, and neonate males and females aged from 33 gestational weeks to 11 months, with 4–7 kidneys per group. Table 1 compiles the characteristics of all donors included in the study. Frozen kidneys were sliced, and the cortical region collected for experiments. Ethics approval and inform consent were handled by IIAA in coordination with each participating ethical committee center and donor.

**TABLE 1:** Characteristics of kidney donors included in the study. Values expressed as mean ± SEM or as percentages.

Adults		Male (7)	Female (4)
Age	Years	51–63	59–63
	Median [Q1 – Q3]	58 [56–60]	62 [60–62]
BMI (kg/m^2^)		31.6 ± 1.8	19.5 ± 1.5
Ethnicity	Caucasian (%)	63	100
	Black/African American (%)	12	0
	Hispano/Latino (%)	25	0
Diabetes (%)		13	0
Hypertension (%)		100	100
Cause of death	Cerebrovascular/stroke (%)	38	75
	Anoxia (%)	62	25
Last eGFR (ml/min/1.73 m^2^)		113.6 ± 8.7	70 ± 10.9
Last blood creatinine value (mg/dL)		0.9 ± 0.1	0.8 ± 0.2
Last blood urea nitrogen value (mg/dL)		27.1 ± 4.1	27.5 ± 5.7
Last urine output (cc/h)		122.1 ± 42.2	107.5 ± 75.2

Neonates		Male (6)	Female (7)
Age		33 gestational weeks to 11 months	37 gestational weeks – 6 months
	Preterm (%)	17	0
Weight (kg)		3.6 ± 0.9	2.8 ± 0.9
Height (cm)		50.2 ± 4.9	44.5 ± 6.3
Ethnicity	Caucasian (%)	83	71
	Hispano/Latino (%)	17	29
Diabetes (%)		0	0
Hypertension (%)		0	0
Cause of death	CPA (%)	0	14
	Head Injury/Child abuse (%)	0	14
	Anoxia	50	0
	Congenital diaphragmatic hernia	17	0
	Cardiac arrest	0	14
	Anencephaly	33	58

### 2.2 Mouse Kidneys

Kidneys from 18.5–23.5 months old male and female C57BL/6N mice were decapsulated and snap‐frozen at −80°C. Pregnant C57BL/6J mice were purchased from Janvier Labs (France), and their offspring were anesthetized with isoflurane and euthanized by exsanguination at 4 weeks of age for kidney collection. Mouse were housed at 22°C–24°C with a relative humidity around 50% during a 12 h/12 h light:dark cycle. Mouse age was selected to correspond to human donor ages (adults) and postnatal kidney maturation state (weaned). Six mice per group were used. Kidneys from six 4.5 months old male and female C57BL/6N mice purchased from Janvier Labs (France) were used to optimize the experimental conditions. Mice were fed the Altromin diet D1310 (0.5% w/w phosphate (0.06% w/w anorganic), 0.7% w/w calcium, 600 IU Vitamin D_3_). For regulatory reasons, C57BL/6N mice were used in this study instead of C57BL/6J. In pilot experiments, cortical BBMV phosphate uptake did not differ between C57BL/6N and C57BL/6J mice of both sexes (data not shown). All experiments were approved by the Zurich Veterinary Office under the reference number ZH089/2023.

### 2.3 SLC34 Inhibitors and IC_50_ Determination

In this study two different inhibitors were used, (1) a highly selective NaPi‐IIa inhibitor, BAY‐767, identified as part of a Bayer Drug Discovery campaign suited to study the pharmacology of selective NaPi‐IIa inhibition in vitro, and (2) LC‐1 a pan‐SLC34 inhibitor previously reported as human NaPi‐IIa inhibitor [22], but found in this study to act as a pan‐SLC34 inhibitor. IC_50_ values (Table S2) were determined for human and mouse transporters using two complementary in vitro assays.

#### 2.3.1 Human SLC34 Inhibition Assay

Inhibition of human NaPi‐IIa, NaPi‐IIb, and NaPi‐IIc was assessed using a membrane potential–based functional assay as previously described (Patent WO 2018/069222 from Bayer AG). NaPi‐IIa and NaPi‐IIb are electrogenic sodium‐dependent phosphate cotransporters, whereas NaPi‐IIc was rendered electrogenic by introduction of three amino acid substitutions (S190A, S192A, S196D), according to [23]. Chinese Hamster Ovary (CHO) T‐Rex cells (Cat. No. R718‐07, Life Technologies) with doxycycline‐inducible expression of human SLC34 transporters were generated and maintained under standard culture conditions (Dulbecco's MEM/F12 (4.5 g/L glucose, Cat. No. 21331–020 Gibco, Germany) supplemented with 2 mM Glutamax, 1.4 mM sodium pyruvate, 20 mM HEPES, 17 mM sodium bicarbonate, 10% Fetal Bovine Serum tetracycline free (Cat. No. 631106, Clontech), 1× Penicillin–Streptomycin (Gibco A5873601), Blasticidin 10 μg/mL and 400 μg/mL Hygromycin). Cells were seeded into 1536‐well plates, induced with doxycycline (0.5 μg/mL), and incubated for 24 h. Transport activity was measured using a fluorescent membrane potential dye (BLUE MPdye, Cat. No. R8034, Molecular devices) following sodium–phosphate–induced depolarization. On the day of the experiment a 1× MPdye Loading Solution was freshly prepared by re‐suspending 15 mg of Blue MPdye powder in 10 mL of NHE buffer sodium‐free (140 mM N‐Methyl‐D‐glucamine, 5.4 mM KCI, 1 mM CaCl_2_, 11 mM D (+) ‐Glucose water fee, 1.2 mM MgCl_2_, 10 mM HEPES; pH 7.4 (adjusted with hydrochloric acid); sterile filtered). Medium (5 μL) was robotically exchanged for sodium‐free NHE buffer, washed twice, then cells were incubated for 5 min at room temperature with 5 μL/well MPdye Loading Solution (1×). Both inhibitors were added to the cells at final test concentrations between 50 μM and 1 nM (0.6 μL/well, final DMSO 0.6%, prepared in MPdye Loading Solution) and incubated for 5 min at room temperature. Plates were analyzed with an in‐house CCD camera device using a λexc 510–545 nm/ λexc 565–625 nm filter. Fluorescence was recorded for 15 s (background M1), then SLC34 was activated by adding 2 μL/well of 30 mM Na^+^/1 mM phosphate, followed by 2–3 min fluorescence recording (M2). Data was normalized to cell number and dye loading efficiency by calculating M2/M1. This quotient was plotted against test compound concentration.

#### 2.3.2 Mouse NaPi‐IIa Inhibition Assay

These assays were performed at NMITT Pharmaservices using the Roboocyte system enabling the fully automated injection of cRNA into *Xenopus laevis* oocytes and the subsequent conductance of automated TEVC measurements [24]. cDNA templates encoding the murine isoform of the NaPi‐IIa transporter were cloned into a vector for oocyte expression and were transcribed into the required cRNAs. Ready‐to‐use *Xenopus* oocytes were purchased from EcoCyte Biosciences. Suitable oocytes were manually selected and placed into 96‐well plates. The oocytes were injected with 1500 ng/μl of cRNA using the Roboocyte automated injection procedure. After injection, the oocytes were kept in Barths solution at 19°C until usage 3–5 days later. Electrophysiological measurements were conducted at room temperature (20°C–24°C) with the Roboocyte system (Multichannel Systems). Measurements were conducted in ND96 buffer (in mM: NaCl 96, KCl 2, CaCl_2_ 1.8, MgCl_2_ 1, HEPES 5, pH 7.4) with different concentrations. The microelectrodes for the Roboocyte were filled with 1.5 M KAc, 1 M KCl, pH 7.2 and had a resistance of 0.2 to 1.5 MΩ.

#### 2.3.3 Data Analysis

Graph Pad Prism or equivalent in house software was used to create sigmoidal dose–response curves (variable slope) and determine IC_50_ values.

#### 2.3.4 SLC34 Inhibitors Handling

The inhibitors were diluted in DMSO to a final stock concentration of 10 mM and stored at −20°C. The inhibitors may be available for research use upon reasonable request.

### 2.4 Functional Assay Into Renal Brush Border Membrane Vesicles (BBMVs)

Renal BBMVs were prepared using the Mg^2+^ precipitation and differential centrifugation method [25]. Uptake of radioactive ^32^P‐phosphate and ^14^C‐L‐leucine (Hartmann analytical GmbH) into BBMVs was assessed using the microfiltration method [26]. 0.125 mM Na_2_HPO_4_/NaH_2_PO_4_ or 0.125 mM L‐leucine stocks containing the corresponding tracer were added into either a sodium‐containing solution (100 mM Mannitol, 20 mM HEPES‐Tris pH 7.4, 125 mM NaCl) or a sodium‐free solution (100 mM Mannitol, 20 mM HEPES‐Tris pH 7.4, 125 mM KCl). Either LC‐1 or BAY‐767 was added to the uptake solutions at the indicated final concentrations; 1% DMSO was used as control. The uptake reaction was performed as described [26] and counts measured using scintillation media (Emulsifier‐Safe, Revvity) and a β‐counter (TriCarb 2900TR Liquid Scintillation Analyzer, Packard Instruments). Sodium‐dependent uptake was calculated as the difference in tracer incorporation between the sodium‐containing and sodium‐free solutions. Leftovers of BBMVs suspensions were snap‐frozen and stored at −80°C for later use in immunoblotting experiments.

#### 2.4.1 Calculations Performed to Obtain Phosphate Uptake Values as Percentage of Control

Uptake of ^32^Pi in the presence of inhibitors (Figure 2) showed the sodium‐dependent component normalized to the sodium‐dependent incorporation in the presence of 1% DMSO and was calculated as follows. For each experiment, 2.5 μL of the ^32^P/0.125 mM Na_2_HPO_4_/NaH_2_PO_4_ solution was added to 4 mL scintillation media (Emulsifier‐Safe, Revvity) and radioactivity (total counts) was measured using a β‐counter (TriCarb 2900TR Liquid Scintillation Analyzer, Packard Instruments) for 30 s. The specific activity was calculated using the equation:

$$\text{Specific activity}\;\left(\text{cpm}/\text{pmol}\right)=\frac{\text{Total counts}\;\left(\text{cpm}\right)-\text{Blank}\;\left(\text{cpm}\right)}{2.5\;\left(\text{μl}\right)*125\;\left(\text{pmol}/\text{μl}\right)}$$

The uptake experiments were performed in triplicate for each sample. The specific activity (S.A.) was then used to calculate the pmol of inorganic phosphate taken up by the BBMVs with the following equation:

$$\text{Pi}\;\left(\text{pmol}\right)=\frac{\text{Average sample counts}\;\left(\text{mtext}\right)-\text{Blank}\;\left(\text{cpm}\right)}{\text{S}.\text{A}.\left(\text{cpm}/\text{pmol}\right)}$$

These values were normalized by protein concentration. BBMVs’ protein concentrations were determined using the DC Protein Assay (Bio‐Rad, Switzerland), following the manufacturer's instructions. For BBMVs prepared from frozen human cortices, the concentrations were between 5 and 12 mg/mL, whereas for BBMVs prepared from frozen mouse kidneys, the concentrations were between 4 and 8 mg/mL. Next, for each sample, sodium‐dependent phosphate uptake in the presence of an inhibitor was calculated as the percentage of the uptake in the presence of the vehicle (1% DMSO), the control.

#### 2.4.2 Calculation of Relative Activity of the Different SLC34 Transporters

The relative contribution of cotransporters to phosphate uptake (Figures 3, 4, 5) was calculated as shown in Figure S2. NaPi‐IIa activity resulted from subtracting the sodium‐dependent phosphate uptake obtained in the presence of the inhibitor BAY‐767 from that of the control. NaPi‐IIb/c related activity was obtained by subtracting the sodium‐dependent phosphate uptake measured upon incubation with the pan‐inhibitor LC‐1 from the uptake in the presence of the inhibitor BAY‐767. Together this represents the SLC34‐related activity. The phosphate uptake remaining after inhibition by LC‐1 accounts for the sodium‐dependent but non‐SLC34‐related activity.

### 2.5 Immunoblotting

Following two 30‐s sonication steps using a Branson Sonifier Cell Disruptor B15 (Emerson Electric), the BBMVs used for the uptake experiments, as well as homogenate preparations from renal tissue, protein concentrations were determined using the Bio‐Rad DC Protein Assay (Cat. No. 5000113–115). A total of 20 μg of either BBMV or homogenate protein was separated on a 9% SDS‐PAGE gel and transferred to a PVDF membrane by electroblotting. Total protein staining was performed using the Revert 700 Total Protein Stain (LI‐COR BioSciences, Part No. 926–11 021) and recorded with the Odyssey CLx imager (LI‐COR Biosciences). After blocking with 5% fat‐free milk, membranes were incubated with the respective primary antibodies overnight at 4°C, and with the corresponding secondary antibody for 2 h at room temperature (RT) (antibodies and dilutions are listed in Table S3). Antibody detection was performed with Immobilon Western Chemiluminescent HRP Substrate (Millipore, Cat. No. WBKLS0500). Images obtained with the Las‐4000 Imaging Analysis System (Fujifilm Medical Systems) were analyzed using Image Studio Lite (LI‐COR Biosciences, USA) and normalized to total protein.

### 2.6 Antibodies Specificity

The antibodies used in this study are listed in Table S3. Rat anti‐NaPi‐IIa, human anti‐NaPi‐IIb, and human anti‐NaPi‐IIc antibodies were custom‐made at Pineda antibody services upon inoculation of antigenic peptides in rabbits and validated in our laboratory by performing Western blots on protein extracts of *Xenopus laevis* oocytes injected with cRNA of the corresponding human cotransporter and/or on BBMVs from native tissue (kidney/intestine) prepared as described in the main body of the manuscript. Anti‐rat NaPi‐IIa antibody (Nterm Rb‐1) was raised against the epitope MSYSERLGGPAVSPLPVRGRH (77% homology to the human sequence). This antibody recognizes NaPi‐IIa in oocytes injected with human NaPi‐IIa cRNA as well as in renal human, mouse, and rat BBMVs (Figure S3A). Anti‐human NaPi‐IIb antibody (644–662 Rb‐1) was raised against the epitope CEDLEEAQEGQDVPVKAPE. This antibody detected a smear in oocytes injected with human NaPi‐IIb cRNA and no signal in water‐injected oocytes (Figure S3B). No signals were observed in intestinal BBMVs from mice and rat. Anti‐human NaPi‐IIc antibody (Cterm Rb‐2) was raised against the epitope CYENPEILASQQL. This antibody recognizes NaPi‐IIc in oocytes injected with human NaPi‐IIc cRNA as well as in renal human and rat BBMVs, but does not recognize the murine NaPi‐IIc (Figure S3C). The specificity of the primary antibody anti‐human PiT‐1 (Cell Signaling, Cat. No. 12765) has been shown previously [27].

### 2.7 Single Cell RNA‐Seq Data Analysis

Previously published human single‐nuclei (GSE183279) [28] and mouse single‐cell (GSE129798) [29] RNA‐seq datasets were processed using Seurat 5.3.0 (R package). Data from healthy human reference controls (*n* = 21) or wildtype adult mice (*n* = 4) only, were analyzed. Datasets were first analyzed combining both sexes, followed by a second analysis where data were stratified by sex. In mouse, we used a pre‐processed dataset, that was filtered for genes/cells (1′000–4′000), mRNA transcripts/cell (1′000–16′000) and mitochondrial genes/cells (< 35%). In human, data was filtered for genes/cells (200–6′000), mRNA transcripts/cell (< 17′000) and mitochondrial genes/cells (< 15%). Normalization, feature selection for highly variable features and data scaling was performed using the *NormalizeData*, *FindVariableFeatures* and *ScaleData* function, respectively. Linear dimensionality reduction with the *RunPCA* function identified useful principal components, used for clustering with the *FindNeighbours* and *FindClusters* functions and for non‐linear dimensionality reduction with the *RunUMAP* function. The ontology of the original dataset was used for cell type annotation. Data was visualized with the *DotPlot* function.

### 2.8 Immunofluorescence

Archived paraffin‐embedded kidney biopsies from healthy kidney donors (7 individuals with mean age of 41.3 ± 16.5 years, 5 males and 2 females, and with a mean eGFR of 111.6 ± 22.9 mL/min/1.73 m^2^) from the Department of Nephropathology at the University of Erlangen‐Nürnberg were automatically deparaffinized with the Pathisto AS‐2 automatic slide stainer. The sections were put in a citrate buffer (sodium citrate 10 mM, pH 6.0) bath for 15 min at sub‐boiling temperatures (88°C‐95°C) and washed in phosphate‐buffered saline (PBS) 3 × 5 min at RT. The slides were treated with 1% SDS/PBS for 5 min, washed with PBS 3 × 5 min, and blocked with 1% bovine serum albumin/PBS for at least 15 min at RT. After blocking, sections were incubated overnight at 4°C with the appropriate primary antibodies against NaPi‐IIa, NaPi‐IIb, NaPi‐IIc, and AQP1 (Table S3). Then samples were washed two times with hypertonic PBS/sodium chloride (NaCl) (18 g NaCl/PBS) and one time with PBS for 5 min each and incubated 1 h at RT with the appropriate secondary antibodies (Table S3) plus 40,6‐diamidine‐20‐phenylindole dihydrochloride (DAPI; Sigma Aldrich, Buchs, Switzerland) 1:1000 diluted in PBSRT. Slides were again washed two times with 18 g NaCl/PBS and one time with PBS for 5 min each. Coverslips were mounted with Glycergel (Dako Cytomation). Fluorescence was detected with a Leica fluorescence microscope (Leica M5500B). The use of Formalin‐Fixed Paraffin‐Embedded material from the archive of the Department of Nephropathology was approved by the Ethics Committee of the Friedrich‐Alexander‐University of Erlangen‐Nürnberg, waiving the need for retrospective consent for the use of archived rest material (Re.‐No. 22–150‐D).

### 2.9 Statistical Analysis

Data analysis was conducted with GraphPad Prism version 10.1.2 (GraphPad Software, USA). No statistical outlier removal was applied in the primary analyses, and no data points were excluded based on their statistical properties. Data exclusion was limited to rare cases of clear technical failure identified through predefined quality control criteria independent of the measured outcome. The data are presented as individual values together with the mean +/− standard error of the mean (SEM). The Two‐way ANOVA followed by Tukey's multiple comparison test or the One‐way ANOVA followed by Dunnet's post hoc test was conducted to compare the groups with significance set at α = 0.05. Normality of model residuals was assessed using the Shapiro–Wilk test together with visual inspection of Q–Q plots, with no evidence of significant deviation from normality observed. Homogeneity of variance was evaluated by inspection of residual distributions and was considered satisfactory for all analyses. Formal a priori power calculations were not performed. Sample sizes were determined by practical and ethical constrains, including the availability of human kidney tissue and predefined experimental designs for animal studies.

## 3 Results

### 3.1 Sodium‐Dependent Phosphate Uptake Into BBMVs Isolated From Human Kidneys

**FIGURE 1:**
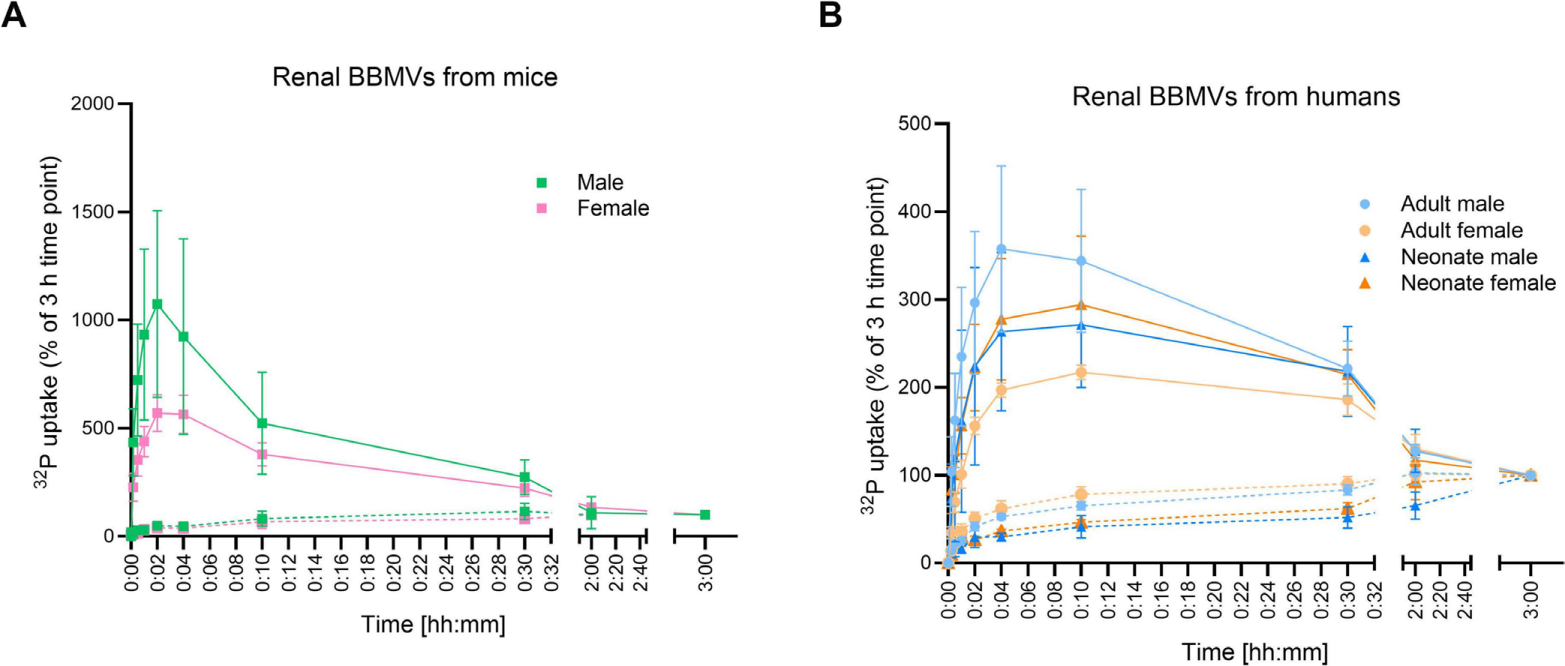
Phosphate uptake into BBMVs isolated from mouse and human kidneys. Time‐course of phosphate uptake into renal BBMVs isolated from (A) kidneys from 4.5 months old male and female C57BL/6N mice and (B) cortices from adult and neonate human kidneys from both sexes. Uptakes were performed in the presence (continuous lines) and absence (dotted lines) of a sodium gradient and were stopped at several time points with a maximal incubation time of 3 h. *N* = 3 samples/group. Graphs represent mean ± SEM.

Phosphate uptake into BBMVs isolated from kidneys of 4.5 months old male and female mice was sodium‐dependent and showed the characteristic overshoot in the presence of a sodium gradient [30, 31] (Figure 1A). Uptakes showed maximal accumulation after 2 min incubation at 25°C and pH 7.4. Using the Mg^2+^ precipitation and differential centrifugation method [25] as for mice, BBMVs were isolated from frozen adult and neonate human renal cortices. Phosphate uptake into BBMVs from human kidneys also showed the characteristic overshoot in the presence of a sodium gradient with maximal accumulation at 4 min for all groups (Figure 1B). Replacing sodium with potassium strongly reduced uptake. These time course experiments demonstrated the feasibility of measuring sodium‐dependent phosphate uptake in BBMVs isolated from frozen human kidneys, and 10 s was determined as the optimal incubation time. This time is in the linear accumulation phase of phosphate transport into the BBMVs from human and murine kidneys.

### 3.2 SLC34 Inhibitors

**FIGURE 2:**
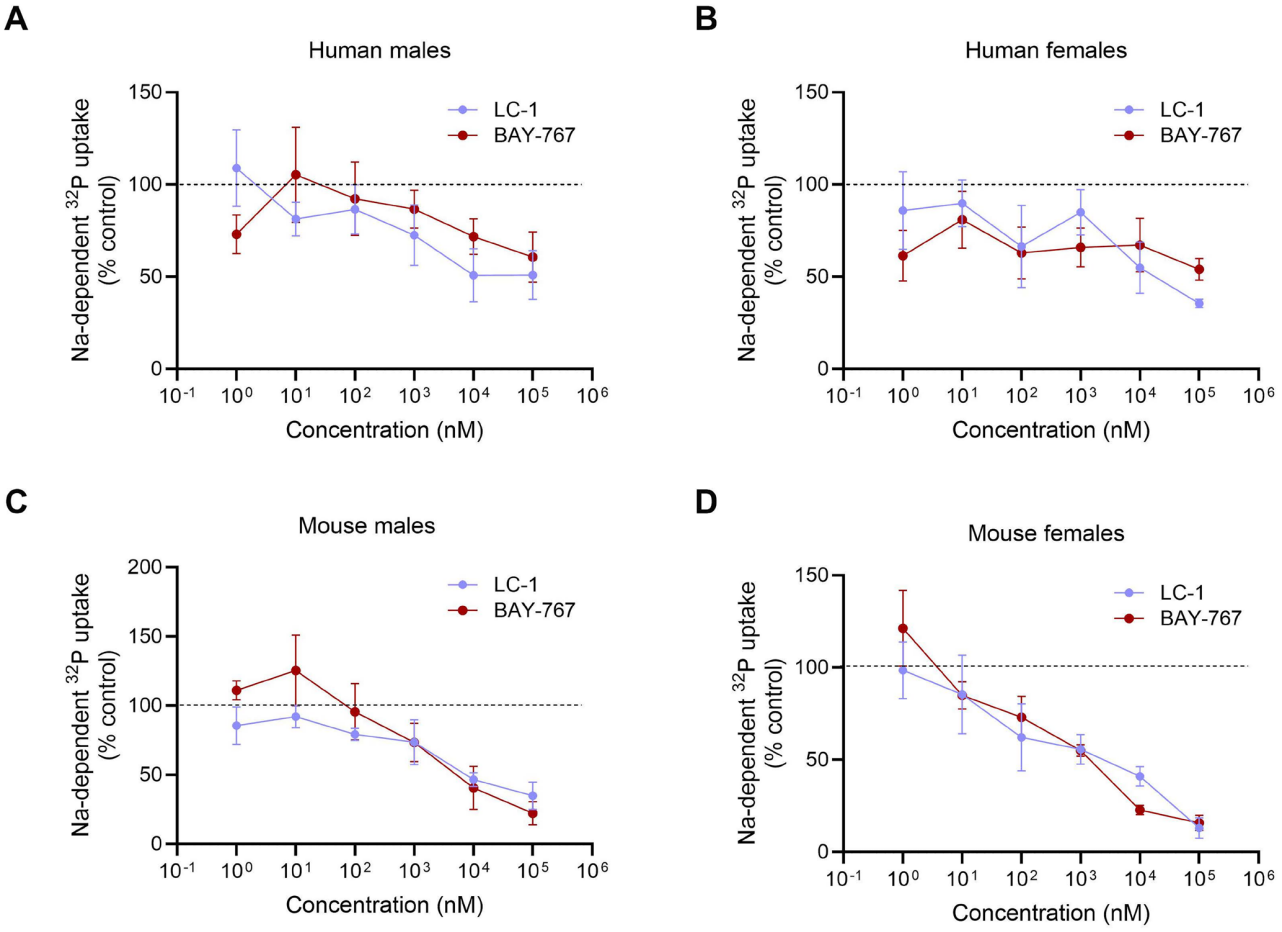
Pharmacological inhibition of sodium‐dependent phosphate uptake into BBMVs isolated from human and mouse kidneys. Phosphate uptakes in the absence and presence of phosphate transport inhibitors were performed in renal BBMVs isolated from (A) adult human males, (B) adult human females, and 4.5 months old C57BL/6N (C) male and (D) female mice. Inhibition of sodium‐dependent phosphate uptake caused by the presence of LC‐1 (a pan‐SLC34 inhibitor; filled circles) and BAY‐767 (the selective NaPi‐IIa inhibitor; open circles) is shown as percentage of control (1% DMSO). Inhibitors were added at final concentrations ranking from 1 nM to 100 μM and uptakes were measured after 10 s incubation. *N* = 3–6 samples/group. Graphs represent mean ± SEM.

Using two in vitro models, mammalian cells and oocytes, we showed that the previously identified compound LC‐1 [22] is a pan‐inhibitor for all three SLC34 family members. The second inhibitor used here, BAY‐767, was identified as a highly selective NaPi‐IIa inhibitor as part of a Bayer Drug Discovery campaign (Table S2). Next, we determined their concentration‐dependent inhibition of SLC34 activity in BBMVs isolated from frozen mouse and human kidneys. Both, LC‐1 and BAY‐767 inhibited sodium‐dependent phosphate uptake into human (Figure 2A,B) and murine (Figure 2C,D) renal BBMVs. Inhibition was concentration‐dependent, with the strongest statistically different effect observed at 100 μM (Figure S4). At concentrations in the uptake solution higher than 100 μM, the inhibitors were not soluble. Sodium‐independent uptake was not affected by either inhibitor (Figure S5). Na^+^‐dependent ^14^C‐L‐Leucine uptake was also unchanged in all experimental groups (Figure S6), further supporting the selectivity of these inhibitors.

### 3.3 The Relative Contribution of Phosphate Cotransporters in Human Kidney Is Age‐Dependent

**FIGURE 3:**
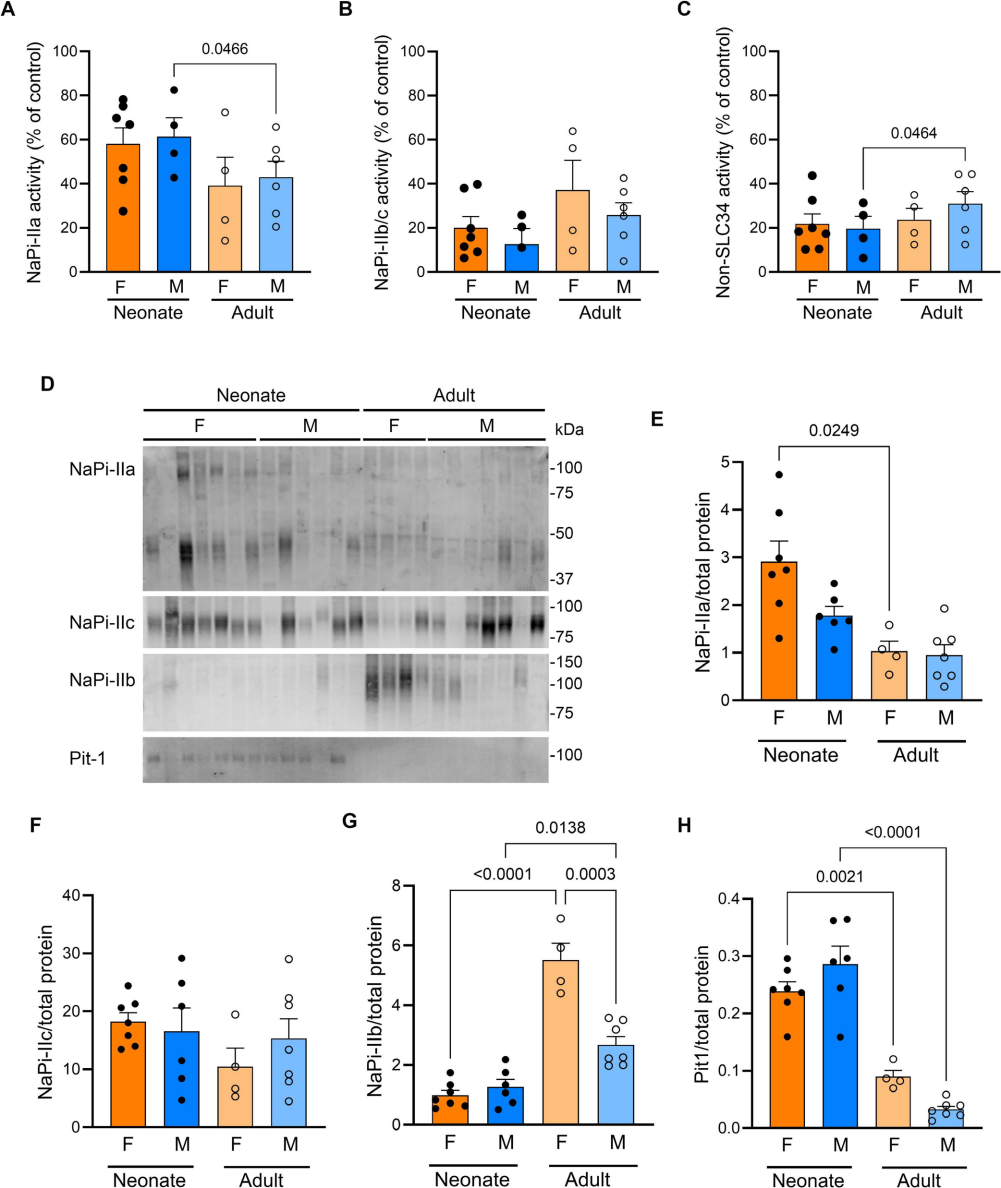
Relative contribution of sodium‐dependent phosphate cotransporters in human kidneys. The relative contribution of (A) NaPi‐IIa, (B) NaPi‐IIb/c and (C) non‐SLC34 cotransporters to renal transport of phosphate was assessed by incubating BBMVs isolated from human renal cortices of neonates and adult female (F) and male (M) donors in the presence of 100 μM of the pan‐inhibitor LC‐1, 100 μM of the selective NaPi‐IIa inhibitor BAY‐767 or 1% DMSO. Uptakes were carried out for 10 s, and relative contributions were calculated as described in the methods and Figure S2. *N* = 6–9 samples/group. Bars represent mean ± SEM. Statistically significant differences were analyzed using Two‐way ANOVA analysis followed by Tukey's multiple comparison test. (D) Representative Western blots of the BBMVs used in the uptake studies (NaPi‐IIa, NaPi‐IIc, NaPi‐IIb) and of protein homogenates (Pit‐1) with the indicated antibodies. (E‐H) Densitometry bar plots of the relative signal intensity of each cotransporter to total protein staining (Figure S8). All bars represent mean ± SEM. Statistically significant differences were analyzed using One‐way ANOVA analysis followed by Dunnet's post hoc test.

The relative contribution of SLC34 transporters to phosphate uptake was calculated as depicted in Figure S2. Phosphate uptake experiments revealed that NaPi‐IIa related activity mediated approximately 60% of the sodium‐dependent uptake of phosphate into renal BBMVs from human neonates of both sexes. This contribution was significantly reduced in adult males compared to neonates (Figure 3A). NaPi‐IIb/c related activity was similar across all groups, accounting for about 20%–40% of total transport (Figure 3B). The contribution of non‐SLC34‐related phosphate transport was about 20% in neonates and increased in males with age (Figure 3C). Validated custom‐made antibodies to detect human SLC34 transporters were used for protein expression level analysis (see Methods and Figure S3). NaPi‐IIa protein levels were higher in neonate females compared to adult females (Figure 3D,E). NaPi‐IIc expression levels did not differ between sexes or with age (Figure 3D,F). In contrast, NaPi‐IIb had higher protein expression levels in adults than in neonates, and female adults showed higher NaPi‐IIb expression levels than adult males (Figure 3D,G). Pit‐1 protein expression levels in renal homogenates were higher in neonates as compared to adults (Figure 3D,H). Pit‐2 protein expression levels could not be analyzed, as we failed to identify specific antibodies (data not shown).

### 3.4 NaPi‐IIa Is the Major Renal Phosphate Transporter in Mice

**FIGURE 4:**
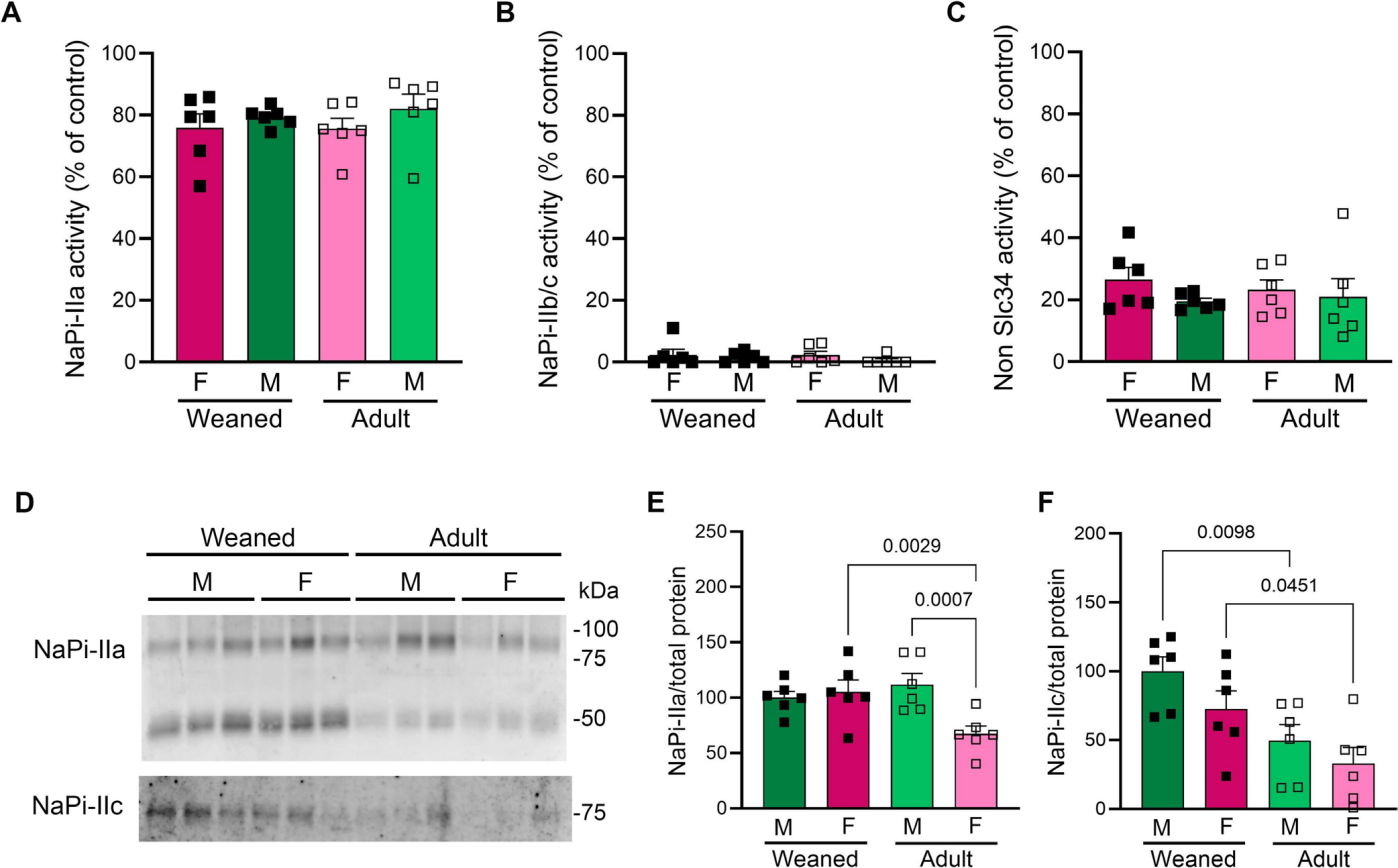
Relative contribution of sodium‐dependent phosphate cotransporters in mouse kidneys. The relative contribution of (A) NaPi‐IIa, (B) NaPi‐IIb/c, and (C) non‐Slc34 cotransporters to renal transport of phosphate was assessed by incubating BBMVs isolated from kidneys of weaned and adult male (M) and female (F) C57BL/6N mice in the presence of 100 μM of the pan‐inhibitor LC‐1, 100 μM of the selective NaPi‐IIa inhibitor BAY‐767, or 1% DMSO. Uptakes were carried out for 10 s, and relative contributions were calculated as described in the methods and Figure S2. *N* = 6 samples/group. Bars represent mean ± SEM. Statistically significant differences were analyzed using Two‐way ANOVA analysis followed by Tukey's multiple comparison test. (D) Representative Western blots of BBMVs used in the uptake studies with antibodies for NaPi‐IIa and NaPi‐IIc. *N* = 6 samples/group distributed in 2 gels. (E‐H) Densitometry bar plots of the relative signal intensity of each cotransporter to total protein staining (Figure S8). All bars represent mean ± SEM. Statistically significant differences were analyzed using Two‐way ANOVA analysis followed by Tukey's multiple comparison test.

No differences between groups were observed in the contribution of the different sodium‐dependent phosphate cotransporters in renal samples from age‐matched (adults) and kidneys matched for maturation stage (weaned) mice (Figure 4A–C). NaPi‐IIa related activity accounted for about 80% of sodium‐dependent phosphate transport in all analyzed groups, while NaPi‐IIb/c related activity contribution was almost negligible, and non‐SLC34 transporters contributed to around 20% of total transport activity. Yet, NaPi‐IIa protein levels were significantly lower in adult female mice when compared to adult male and weaned females (Figure 4D,E), whereas NaPi‐IIc expression levels were higher in weaned mice (Figure 4D,F). Pit‐1 was not detectable by immunoblotting in murine BBMVs.

### 3.5 Differences in the Relative Contribution of Sodium‐Dependent Phosphate Cotransporters in Mice and Humans

**FIGURE 5:**
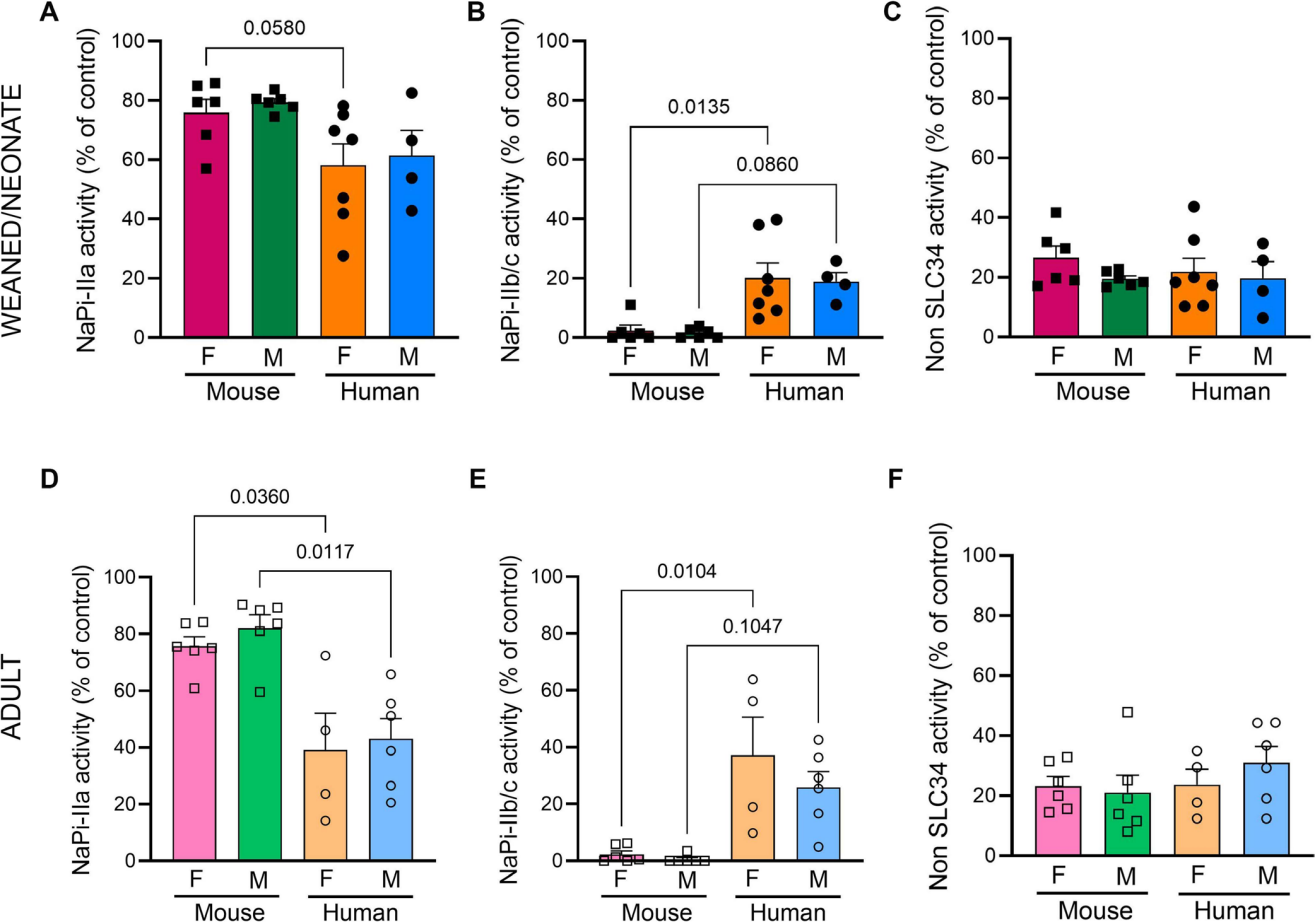
Comparison of the relative contribution of the sodium‐dependent phosphate cotransporters in human and age‐matched mice. Comparison of relative contributions of (A, D) NaPi‐IIa, (B, E) NaPi‐IIb/c, and (C, F) non‐SLC34 cotransporters to transport of phosphate in BBMVs isolated from kidneys of neonate/weaned and adult humans and mice. Graphs are based on data presented in Figures 3 and 4. *N* = 6–9 samples/group. Bars represent mean ± SEM. Statistically significant differences were analyzed using one‐way ANOVA analysis followed by Dunnet's post hoc test.

Clear differences were observed in the relative contribution of the sodium‐dependent cotransporters when comparing mice and humans. Neonate human females showed lower NaPi‐IIa relative contribution (60%) to sodium‐dependent phosphate transport than kidney maturation‐matched (weaned) female mice (80%), almost reaching significance (Figure 5A). NaPi‐IIb/c relative contribution was higher in neonate humans (20%) compared to kidney maturation stage matched (weaned) mice (negligible), though it reached significance only in females (Figure 5B), whereas the contribution of non‐SLC34 transporter to sodium‐dependent phosphate transport was similar (20%) in all groups. NaPi‐IIa related relative contribution was lower in adult humans (40%) than in age‐matched mice (80%) (Figure 5D). Instead NaPi‐IIb/c related activity tended to be higher in adult humans (20%–30%) compared to age‐matched mice (negligible), though significance was only detected in females (Figure 5E). In adults, the contribution of non‐SLC34 transporter to sodium‐dependent phosphate transport was around 30% with no sex or species differences (Figure 5F). These results indicate clear differences in the relative contribution of the sodium‐dependent phosphate transporters to renal phosphate reabsorption between humans and mice, with sex influencing some of these differences. Changes were more evident in adults compared with younger individuals.

### 3.6 Human Kidney scRNA‐Seq and Cellular Protein Expression

**FIGURE 6:**
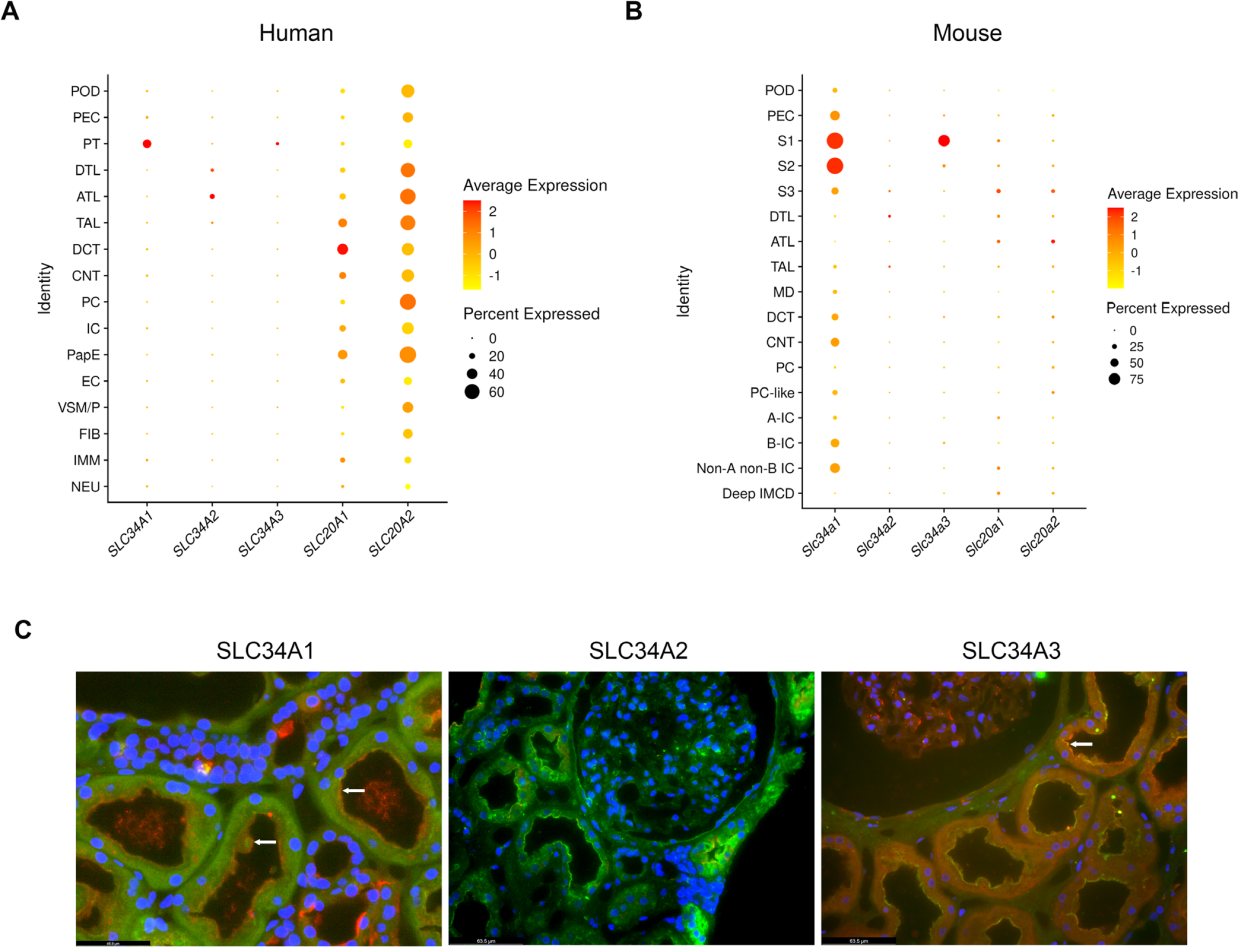
mRNA expression of sodium‐phosphate cotransporter paralogues in kidney. Public single nuclei and single cell RNA transcriptome data from (A) human and (B) murine kidney, respectively, was analyzed. POD = Podocyte, PEC = Parietal Epithelial Cell, PT = Proximal Tubule, S1 = Proximal Tubule Segment 1, S2 = Proximal Tubule Segment 2, S3 = Proximal Tubule Segment 3, DTL = Descending Thin Limb, ATL = Ascending Thin Limb, TAL = Thick Ascending Limb, MD = Macula Densa, DCT = Distal Convoluted Tubule, CNT = Connecting Tubule, PC = Principal Cell, PC‐like = Principal‐like Cell, IC = Intercalated Cell, IC‐A = Intercalated‐A Cell, IC‐B = Intercalated‐B Cell, IC non‐A non‐B = Non‐A Non‐B Intercalated Cell, Deep IMCD = Deep Inner Medullary Collecting Duct, PapE = Papillary Tip Epithelial Cell. Transcriptome data for human and mice males and females are displayed in Figure S7. (C) Immunolocalization of SLC34A1, SLC34A2 and SLC34A3 in human kidney biopsies. NaPi‐IIa/b/c in red, AQP1 in green, DAPI blue, original magnification 400 x. White arrows indicate localization of SLC34 transporters.

We analyzed publicly available single cell and single nuclei transcriptome data from murine and human adult kidney, respectively [28, 29] to corroborate functional and immunoblotting data (Figure 6A,B and Figure S7). *SLC34A1* and *SLC34A3* transcripts were both expressed in proximal tubule (PT) of human adult kidney with little expression in other cell types, while *SLC34A2* was mostly found in ascending and descending thin limbs of the loop of Henle (ATL and DTL). *SLC20A1* and *SLC20A2* mRNAs were expressed along the entire nephron. In mouse kidney, *Slc34a1* mRNA expression was mostly detected in S1 and S2 portions of proximal tubule but also found in other segments while *Slc34a3* was restricted to proximal tubule. In mice, *Slc20a1* and *Slc20a2* were expressed along the entire nephron albeit at very low levels. Protein localization of SLC34 paralogues has been reported in rodent but not in human kidney. Here, we performed immunofluorescence on kidney biopsies from healthy kidney donors and found SLC34A1 and SLC34A3 but not SLC34A2 localized in the BBM of proximal tubules, which were identified by positive staining with AQP1 (Figure 6C). No specific staining was detected with the PiT‐1 antibody available to us (data not shown).

## 4 Discussion

Renal phosphate handling is critical for maintaining and controlling systemic phosphate balance [1]. Several sodium‐phosphate cotransporters belonging to the SLC20 and SCL34 families have been identified in the kidney. Even though the first renal phosphate cotransporters were cloned more than 25 years ago, their relative contribution to renal phosphate handling in humans has never been addressed [2]. Animal experiments with genetic deletion of *Slc34a1* (NaPi‐IIa) or *Slc34a3* (NaPi‐IIc) indicate a predominant role for NaPi‐IIa [4, 10, 32]. In contrast, the discovery of loss‐of‐function *SLC34A1* and *SLC34A3* variants in humans suggests a distinct relevance based on the clinical manifestations in these patients [13, 15, 17, 33, 34]. Our data provide now the first direct evidence for the relative contribution of different phosphate transporters to phosphate transport by the proximal tubule BBM in humans, uncover species differences, and show distinct age‐related changes.

Renal reabsorption of phosphate occurs mostly in the proximal tubule as demonstrated by various micropuncture studies in different animal models [35]. There is limited evidence indicating an additional minor phosphate reabsorption in nephron segments beyond the proximal tubule. Mechanisms of proximal tubular phosphate reabsorption have been extensively characterized first in micropuncture studies and later in isolated BBMVs experiments. Collectively, these studies showed a strict dependence of phosphate transport on sodium, modulation by extracellular (luminal) pH, and evidence for electroneutral and electrogenic components [2, 3, 35]. The later cloning of *SLC34A1* (NaPi‐IIa), *SLC34A3* (NaPi‐IIc), and *SLC20A2* (PiT‐2) and their functional characterization in heterologous expression systems closely resembled these features. Also, studies in gene‐modified animals showed that radiotracer fluxes in BBMVs can reflect specific transporters and elucidate their relevance and role [5, 25, 36]. Thus, the classic BBMV technique is a useful tool to further characterize renal phosphate transport in human kidney.

Most studies examining substrate transport in renal BBMVs used kidneys collected from mice, rats, or rabbits while only a few studies used human kidney [37, 38, 39, 40, 41]. Nevertheless, the latter studies demonstrated feasibility and similar properties of BBMVs as those obtained from rodent kidneys. Here, we obtained human kidneys that were considered not suitable for transplant, mostly because of vascular abnormalities. We selected only kidneys where renal function parameters were in the normal range before donation, and fibrosis and kidney injury markers were absent or showed low protein expression levels, and where donors had no evidence for an acute SARS‐CoV‐2 infection or other conditions that could severely affect kidney function. Warm and cold ischemia time was limited to a total of less than 12 h. Kidneys from neonates were mostly obtained from neonates with severe brain malformation that led to death shortly after birth. Other genetic defects, where known, were excluded. BBMVs obtained from frozen human kidneys showed the same overshoot of ^32^P accumulation, similar ^14^C‐leucine uptake, sodium‐dependence, and equilibration after 2 h as BBMVs isolated from frozen mouse kidneys and published for other species, demonstrating integrity and functionality of the human BBMV preparations.

We used two novel inhibitors for SLC34 cotransporters that were selected because of their specificity for NaPi‐IIa (BAY‐767) and their ability to inhibit all SLC34 paralogues (LC‐1), allowing us to differentiate the relative contribution of NaPi‐IIa, NaPi‐IIb/c, and non‐SLC34 cotransporters. A distinct contribution of NaPi‐IIb and NaPi‐IIc could not be determined due to the lack of specific inhibitors for these transporters. The observed non‐SLC34 relative contribution possibly reflects SLC20 mediated phosphate transport, even though other unknown transporters cannot be ruled out. Unfortunately, no specific inhibitors of SLC20 transporter activity are known to further test this hypothesis [42].

Our experiments resulted in several notable observations. First, we were able to distinguish the three sodium‐dependent phosphate cotransporter activities, i.e., NaPi‐IIa‐, NaPi‐IIb/c‐, and non‐SLC34‐related. The presence of these activities is in agreement with the reported expression of NaPi‐IIa and NaPi‐IIc as well as PiT‐2 in renal proximal tubules. Immunohistochemistry has localized all three proteins to the BBM of proximal tubules in rodent kidney [5, 43, 44]. Yet, single cell nuclei transcriptomics from murine kidneys confirmed robust expression of the *Slc34a1 and Slc34a3*, but very low *Slc20a1* and *Slc20a2* expression in adult murine proximal tubule cells. Immunofluorescence on human kidney demonstrates here that NaPi‐IIa and NaPi‐IIc are indeed detectable in the BBM of proximal tubules in adult kidney matching transport activities in BBMVs. The expression of NaPi‐IIb in proximal tubule is not detectable by immunohistochemistry in murine [12] and human neonate kidney (here), but is detectable in human adult kidney with higher expression in females. At mRNA levels the expression appears to be very low.

The second major and important observation is the difference in functional activities between murine and human kidneys. Our data explains some of the discrepancies on the relative relevance of transporter paralogues observed between murine and human studies. NaPi‐IIa related activity dominated in murine kidneys at both ages and in both sexes, while NaPi‐IIc related activity was negligible. About 20% transport activity was not inhibited by SLC34 inhibitors and is possibly mediated by SLC20 paralogues. Instead, in human kidneys, NaPi‐IIa activity was less prominent, particularly in adults, and we detected NaPi‐IIb/c related activity (probably mostly NaPi‐IIc), as well as a residual activity, which may be due to SLC20 transporters. Importantly, NaPi‐IIa related contribution declined with age, while NaPi‐IIb/c activity persisted in adult kidneys consistent with the persistent hyperphosphaturia and hypophosphatemia in carriers of SLC34A3 mono‐ and biallelic loss‐of‐function variants [15, 17].

Finally, there is also some evidence for sex‐dependent differences in transporter activities and expression in our data. In humans, sex differences in serum phosphate and TmP/GFR appear to be more pronounced in men and women beyond the age of around 40 years [19, 45]. Whether the differences observed in our experiments are sufficient to explain variances between sexes remains to be further examined. In mice, female sex hormones have been shown to suppress expression of renal phosphate transporters [46]. There is no such data available for humans, and we do not know the state of our kidney donors' sex hormones, although we can assume that most women were postmenopausal. However, differences in glomerular filtration rate and in endocrine regulators of renal phosphate handling may also play an important role in explaining some sex differences.

Our data may have implications for the development of drugs specifically inhibiting renal phosphate reabsorption [6, 7, 42, 47]. The important contribution of NaPi‐IIa and NaPi‐IIb/c related activities suggests that inhibitors that can target NaPi‐IIa and NaPi‐IIc may be more powerful than blocking only NaPi‐IIa. Recent studies using NaPi‐IIa specific inhibitors in mice and rats suggested that in these species blocking renal phosphate reabsorption is a promising and novel approach to reduce phosphate overload in the context of CKD, and its endocrine and cardiovascular consequences. In humans, dual blockade of NaPi‐IIa and NaPi‐IIc may provide better results.

## 5 Conclusion

In conclusion, we provide the first data on the contribution of different sodium‐dependent phosphate cotransporter activities in human kidneys and show distinct and relevant differences between humans and mice. These differences call for caution in interpreting data from animal experiments. There are also age‐dependent differences that may explain some of the age‐dependent clinical manifestations in patients with *SLC34A1* and *SLC34A3* variants. These data are relevant for choosing targets for pharmacological blockade of renal phosphate reabsorption.

## Author Contributions

**Anja Giese:** conceptualization, methodology, writing – review and editing. **Jürgen Klar:** conceptualization, methodology, writing – review and editing. **Alexander Ehrmann:** conceptualization, methodology, writing – review and editing. **Isabel Rubio‐Aliaga:** conceptualization, visualization, writing – original draft, writing – review and editing, supervision, project administration. **Ashley L. Fernandes:** investigation, writing – review and editing, visualization, methodology. **Laurine Lang:** investigation, writing – original draft, methodology, visualization, formal analysis. **Rupert Busch:** investigation, writing – review and editing. **Hannah Auwerx:** investigation, writing – review and editing. **Carsten A. Wagner:** conceptualization, funding acquisition, writing – original draft, writing – review and editing, supervision, project administration. **Christoph Daniel:** writing – review and editing, methodology. **Nati Hernando:** supervision, writing – review and editing, conceptualization.

## Funding

Schweizerischer Nationalfonds zur Förderung der Wissenschaftlichen Forschung, 212303.

## Conflicts of Interest

C.A.W. reports honoraria from Kyowa Kirin. J.K. A.G. and A.E. are employees of the Bayer AG, Germany and authors of the patent application WO 2018/069222 disclosing BAY‐767. All other authors declare that they are not aware of any conflicts of interest, and that the results presented in this paper have not been published previously in whole or part.

## Supporting information


**Figure S1:** COL1A1, NGAL and KIM‐1 protein expression levels in human kidneys. Representative Western blots of protein homogenates from non‐transplantable human kidneys acquired from the International Institution for the Advancement of Medicine (IIAM, USA). COL1A1 shows a band at 220 kDa, NGAL at 22 kDa and KIM‐1 shows two bands at 50 and 100 kDa (glycosylated protein).
**Figure S2:** Calculation of the relative contributions of SLC34 transporters to total Na+‐dependent phosphate uptake. Sodium‐dependent phosphate uptake into BBMVs was expressed as percentage of uptake in the control condition (1% DMSO, vehicle), as described in the methods section. The relative contribution of NaPi‐IIa to renal phosphate uptake was calculated by subtracting the uptake in the presence of 100 μM of the specific NaPi‐IIa inhibitor (BAY‐767) from the control. The relative activity of NaPi‐IIb/c was determined by subtracting the uptake measured with 100 μM of the SLC34‐pan inhibitor LC‐1 from that measured in the presence of 100 μM of BAY‐767. The uptake measured in the presence of LC‐1 reflects the non‐SLC34‐related activity. Together, the contributions of NaPi‐IIa and NaPi‐IIb/c represent the SLC34‐dependent phosphate transport.
**Figure S3:** Antibodies specificity. Representative immunoblots of antibody specificity testing experiments. (A) Anti‐rat NaPi‐IIa (Nterm Rb‐1) testing with renal BBMVs from wildtype (lanes 1–2) and NaPi‐IIa knockout mice (lanes 3–4) and rat renal BBMVs (lanes 5–6), human renal BBMVs (lanes 7–10), lysates of oocytes injected with water (lane 11) and lysates of oocytes injected with human NaPi‐IIa cRNA (lanes 12–13). (B) Anti‐human NaPi‐IIb (644–662 Rb‐1) testing with ileal BBMVs from wildtype (lanes 1–2) and NaPi‐IIb knockout [1] mice (lanes 3–4) and rat duodenal BBMVs (lanes 5–6), lysates of oocytes injected with water (lane 7) and lysates of oocytes injected with human NaPi‐IIb cRNA (lane 8). (C) Anti‐human NaPi‐IIc (Cterm Rb‐2) testing with renal BBMVs from wildtype (lanes 1–2) and NaPi‐IIc knockout [2] mice (lanes 3–4), rat renal BBMVs (lanes 5–6), human renal BBMVs (lanes 7), human renal BBMVs (lanes 8–9), lysates of oocytes injected with water (lane 10) and lysates of oocytes injected with human NaPi‐IIc cRNA (lane 11). The red arows indicate the signals of the proteins of interest.
**Figure S4:** Sodium‐dependent phosphate uptake into renal BBMVs in the presence of 100 μM of the inhibitors LC‐1 and BAY‐767. Uptake of phosphate into BBMVs isolated from renal cortices of adult human males (A) and females (B) and from kidneys from 4.5 months old male (C) and female (D) mice. Uptake was determined in the presence of a sodium gradient and in the absence (only 1% DMSO, vehicle present) or presence of 100 μM of either LC‐1 or BAY‐7676. N = 3–6 samples/group. All bars represent mean ± SEM. Statistical significant differences were analyzed using One‐way ANOVA analysis followed by Dunnet's post hoc test.
**Figure S5:** Sodium‐independent phosphate uptake into renal BBMVs in the presence of the inhibitors LC‐1 and BAY‐767. Uptake of phosphate into BBMVs isolated from renal cortices of adult human males (A) and females (B) and from kidneys from 4.5 months old male (C) and female (D) mice. Uptake was determined in the absence of a sodium gradient and in the absence (1% DMSO, vehicle) or presence of 100 μM of either LC‐1 or BAY‐7676. N = 3–7 samples/group. All bars represent mean ± SEM. No statistically significant differences were observed using One‐way ANOVA analysis.
**Figure S6:** Na+‐dependent leucine uptake in all experimental groups. Uptake of 14C‐leucine into BBMVs isolated from renal cortices from human neonate males (A) and females (B), and human adult males (C) and females (D) as well as from kidneys from weaned male (E) and female (F) C57BL/6J mice and adult male (G) and female (H) C57BL/6N mice. Uptakes were determined in the absence and presence of a sodium gradient and in the absence (1% DMSO, vehicle) or presence of 100 μM of either LC‐1 or BAY‐7676. N = 4–6 samples/group. All bars represent mean ± SEM. Statistical differences were analyzed using One‐way ANOVA analysis followed by Dunnet's post hoc test.
**Figure S7:** Sex‐specific single nuclei and single cell transcriptome data from murine and human kidney. Expression of SLC34A1, SLC34A2, SLC34A3, SLC20A1, and SLC20A2 in the human single‐nuclei (GSE183279) [3] and mouse single‐cell (GSE129798) [4] RNA‐seq datasets were stratified by sex category. POD = Podocyte, PEC = Parietal Epithelial Cell, PT = Proximal Tubule, S1 = Proximal Tubule Segment 1, S2 = Proximal Tubule Segment 2, S3 = Proximal Tubule Segment 3, DTL = Descending Thin Limb, ATL = Ascending Thin Limb, TAL = Thick Ascending Limb, MD = Macula Densa, DCT = Distal Convoluted Tubule, CNT = Connecting Tubule, PC = Principal Cell, PC‐like = Principal‐like Cell, IC = Intercalated Cell, IC‐A = Intercalated‐A Cell, IC‐B = Intercalated‐B Cell, IC non‐A non‐B = Non‐A Non‐B Intercalated Cell, Deep IMCD = Deep Inner Medullary Collecting Duct, PapE = Papillary Tip Epithelial Cell, EC = Endothelial Cell, VSM/p = Vascular Smooth Muscle Cell/Pericyte, FIB = Fibroblast, IMM = Immune Cell, NEU = Neural Cell.
**Figure S8:** Total protein staining. Total protein staining images from immunoblotting analysis of (A) human NaPi‐IIa, (B) human NaPi‐IIc, (C) human NaPi‐IIb, (D) human Pit1, (E) mouse NaPi‐IIa and (F) mouse NaPi‐IIc.
**Table S1:** Renal suitability scores used to determine eligibility of kidneys for study inclusion. Estimated glomerular filtration rate (eGFR) was calculated using the CKD‐EPI 2021 equation (6) from hospitalization (initial) to kidney donation (last). For initial and last eGFR, scores were assigned as follows: 0 if eGFR ≥ 60 mL/min/1.73 m2; 1 if eGFR < 60 mL/min/1.73 m2. EGFR decline was expressed as a percentage and calculated as (initial eGFR − last eGFR)/initial eGFR × 100. Scores for eGFR decline were assigned as: 0 for no decline or initial eGFR ≥ 60 mL/min/1.73 m2; −1 for initial eGFR < 60 mL/min/1.73 m2 and last eGFR ≥ 60 mL/min/1.73 m2; 1 for ≤ 10% decline; and 2 for > 10% decline. Fibrosis and kidney injury scores were assessed by densitometric analysis of COL1A1, NGAL, and KIM‐1 protein signals, normalized to total protein in Western blot images (Figure S1). Scores for fibrosis were assigned as: 0 for COL1A1 densitometry values ≥ 0 and < 1.5; 1 for ≥ 1.5 and ≤ 2 values; and 2 for > 2 values. Scores for kidney injury for NGAL were assigned as: 0 for densitometry values ≥ 0 and < 5; 1 for ≥ 5 and ≤ 10 values; and 2 for > 10 values. Scores for kidney injury for KIM‐1 were assigned as: 0 for densitometry values ≥ 0 and < 0.7; 1 for ≥ 0.7 and ≤ 0.8 values; and 2 for > 0.8 values. Total score is the sum of the score for each individual, and classified as suitable (light green, score: 0–2), unsuitable (light red, score > 2). M. Male, F: female.
**Table S2:** IC_50_ of SLC34 inhibitors in vitro.
**Table S3:** Antibodies.

## Data Availability

The data that support the findings of this study are available on request from the corresponding author. The data are not publicly available due to privacy or ethical restrictions.
