# Supplementary material for: Renal Phosphate Reabsorption in Humans Depends on at Least Three Distinct Transporters Unlike in Mice
Source: Acta Physiol (Oxf). 2026 Jul 23;242(8):e70271. doi: 10.1111/apha.70271 (PMC13396815; doi:10.1111/apha.70271)
Supplement: Supplementary file 1 — Figure S1: COL1A1, NGAL and KIM‐1 protein expression levels in human kidneys. Representative Western blots of protein homogenates from non‐transplantable human kidneys acquired from the International Institution for the Advancement of Medicine (IIAM, USA). COL1A1 shows a band at 220 kDa, NGAL at 22 kDa and KIM‐1 shows two bands at 50 and 100 kDa (glycosylated protein). Figure S2: Calculation of the relative contributions of SLC34 transporters to total Na+‐dependent phosphate uptake. Sodium‐dependent phosphate uptake into BBMVs was expressed as percentage of uptake in the control condition (1% DMSO, vehicle), as described in the methods section. The relative contribution of NaPi‐IIa to renal phosphate uptake was calculated by subtracting the uptake in the presence of 100 μM of the specific NaPi‐IIa inhibitor (BAY‐767) from the control. The relative activity of NaPi‐IIb/c was determined by subtracting the uptake measured with 100 μM of the SLC34‐pan inhibitor LC‐1 from that measured in the presence of 100 μM of BAY‐767. The uptake measured in the presence of LC‐1 reflects the non‐SLC34‐related activity. Together, the contributions of NaPi‐IIa and NaPi‐IIb/c represent the SLC34‐dependent phosphate transport. Figure S3: Antibodies specificity. Representative immunoblots of antibody specificity testing experiments. (A) Anti‐rat NaPi‐IIa (Nterm Rb‐1) testing with renal BBMVs from wildtype (lanes 1–2) and NaPi‐IIa knockout mice (lanes 3–4) and rat renal BBMVs (lanes 5–6), human renal BBMVs (lanes 7–10), lysates of oocytes injected with water (lane 11) and lysates of oocytes injected with human NaPi‐IIa cRNA (lanes 12–13). (B) Anti‐human NaPi‐IIb (644–662 Rb‐1) testing with ileal BBMVs from wildtype (lanes 1–2) and NaPi‐IIb knockout [1] mice (lanes 3–4) and rat duodenal BBMVs (lanes 5–6), lysates of oocytes injected with water (lane 7) and lysates of oocytes injected with human NaPi‐IIb cRNA (lane 8). (C) Anti‐human NaPi‐IIc (Cterm Rb‐2) testing with renal BBMVs from wildty [file APHA-242-e70271-s001.docx]

**SUPPLEMENTARY MATERIAL**

**Fernandes AL & Lang L et al.** **Renal phosphate reabsorption in humans depends on at least three distinct transporters unlike in mice**

**SUPPLEMENTARY FIGURES**


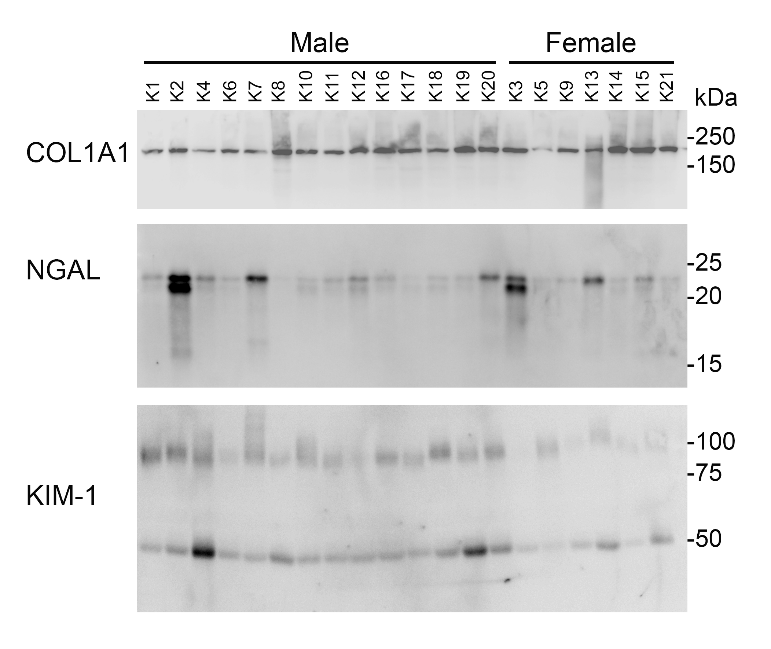


**Supplementary Figure S1.** **COL1A1, NGAL and KIM-1 protein expression levels in human kidneys.** Representative Western blots of protein homogenates from non-transplantable human kidneys acquired from the International Institution for the Advancement of Medicine (IIAM, USA). COL1A1 shows a band at 220 kDa, NGAL at 22 kDa and KIM-1 shows two bands at 50 and 100 kDa (glycosylated protein).


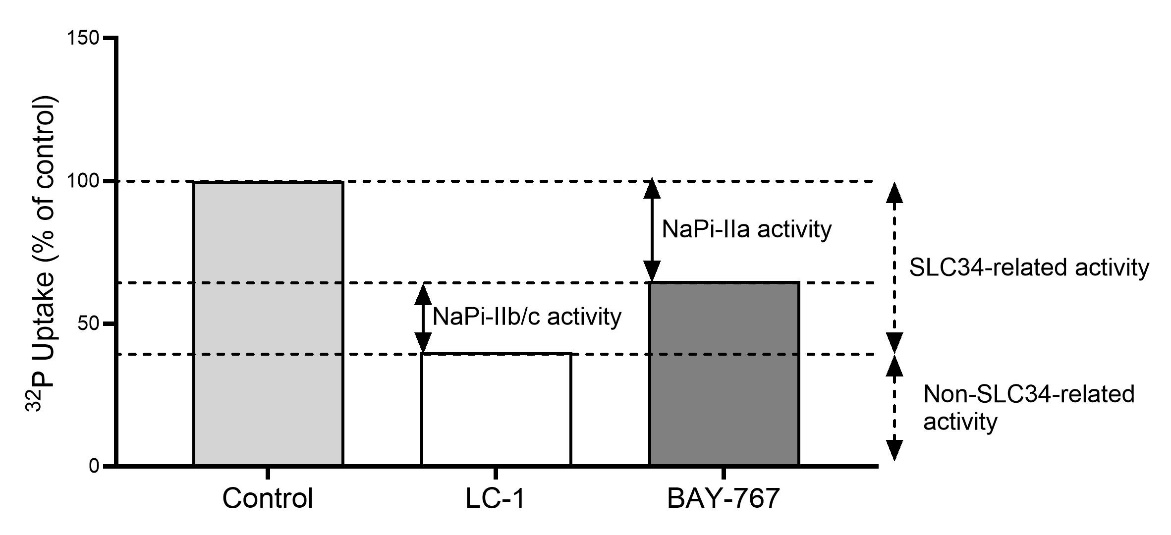


**Supplementary Figure S2.** **Calculation of the relative contributions of SLC34 transporters to total Na^+^-dependent phosphate uptake.** Sodium-dependent phosphate uptake into BBMVs was expressed as percentage of uptake in the control condition (1% DMSO, vehicle), as described in the methods section. The relative contribution of NaPi-IIa to renal phosphate uptake was calculated by subtracting the uptake in the presence of 100 μM of the specific NaPi-IIa inhibitor (BAY-767) from the control. The relative activity of NaPi-IIb/c was determined by subtracting the uptake measured with 100 μM of the SLC34-pan inhibitor LC-1 from that measured in the presence of 100 μM of BAY-767. The uptake measured in the presence of LC-1 reflects the non-SLC34-related activity. Together, the contributions of NaPi-IIa and NaPi-IIb/c represent the SLC34-dependent phosphate transport.

**
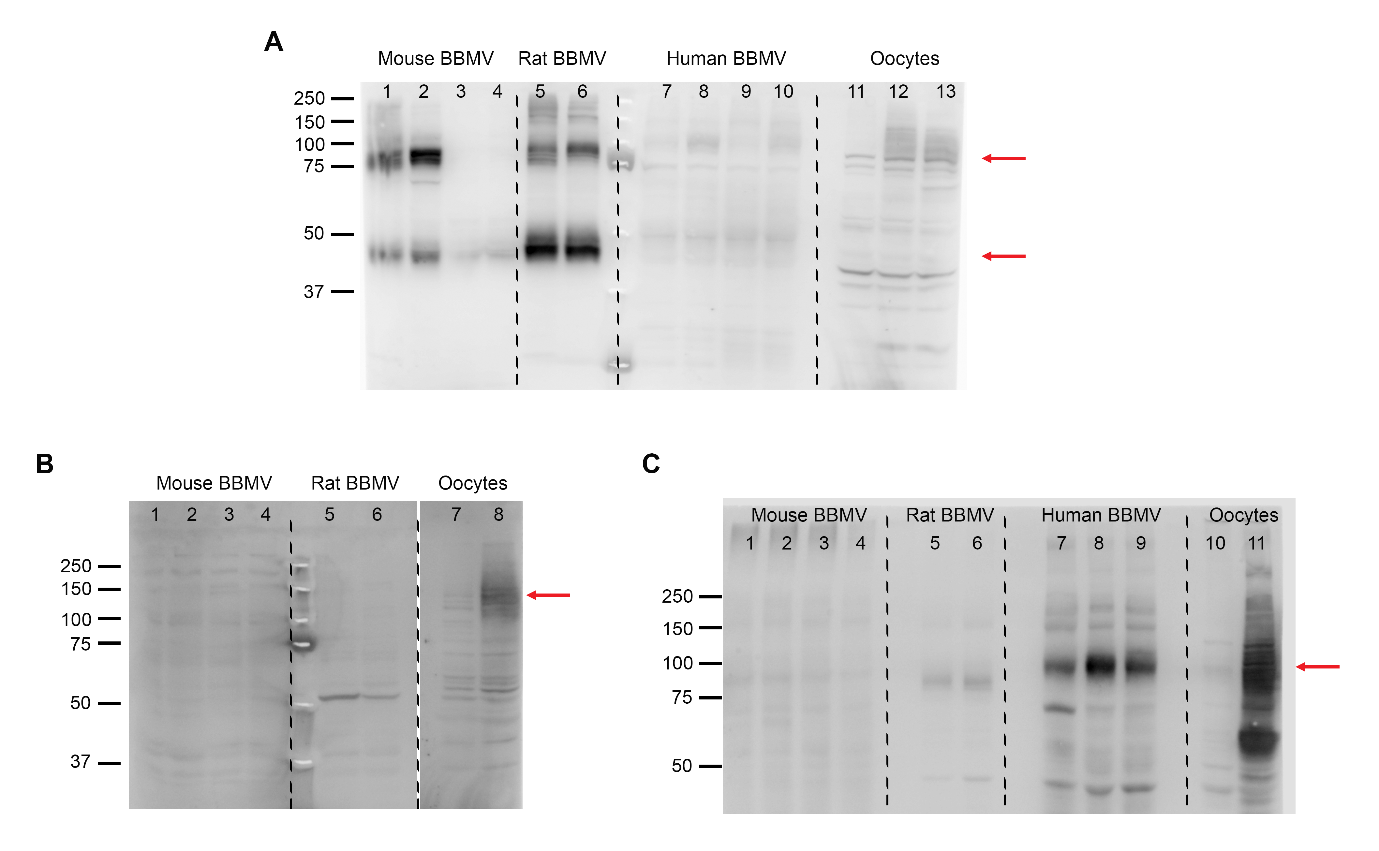
**

**Supplementary Figure S3. Antibodies specificity**. Representative immunoblots of antibody specificity testing experiments. (A) Anti-rat NaPi-IIa (Nterm Rb-1) testing with renal BBMVs from wildtype (lanes 1-2) and NaPi-IIa knockout mice (lanes 3-4) and rat renal BBMVs (lanes 5-6), human renal BBMVs (lanes 7-10), lysates of oocytes injected with water (lane 11) and lysates of oocytes injected with human NaPi-IIa cRNA (lanes 12-13). (B) Anti-human NaPi-IIb (644-662 Rb-1) testing with ileal BBMVs from wildtype (lanes 1-2) and NaPi-IIb knockout ^1^ mice (lanes 3-4) and rat duodenal BBMVs (lanes 5-6), lysates of oocytes injected with water (lane 7) and lysates of oocytes injected with human NaPi-IIb cRNA (lane 8). (C) Anti-human NaPi-IIc (Cterm Rb-2) testing with renal BBMVs from wildtype (lanes 1-2) and NaPi-IIc knockout ^2^ mice (lanes 3-4), rat renal BBMVs (lanes 5-6), human renal BBMVs (lanes 7), human renal BBMVs (lanes 8-9), lysates of oocytes injected with water (lane 10) and lysates of oocytes injected with human NaPi-IIc cRNA (lane 11). The red arows indicate the signals of the proteins of interest.

**
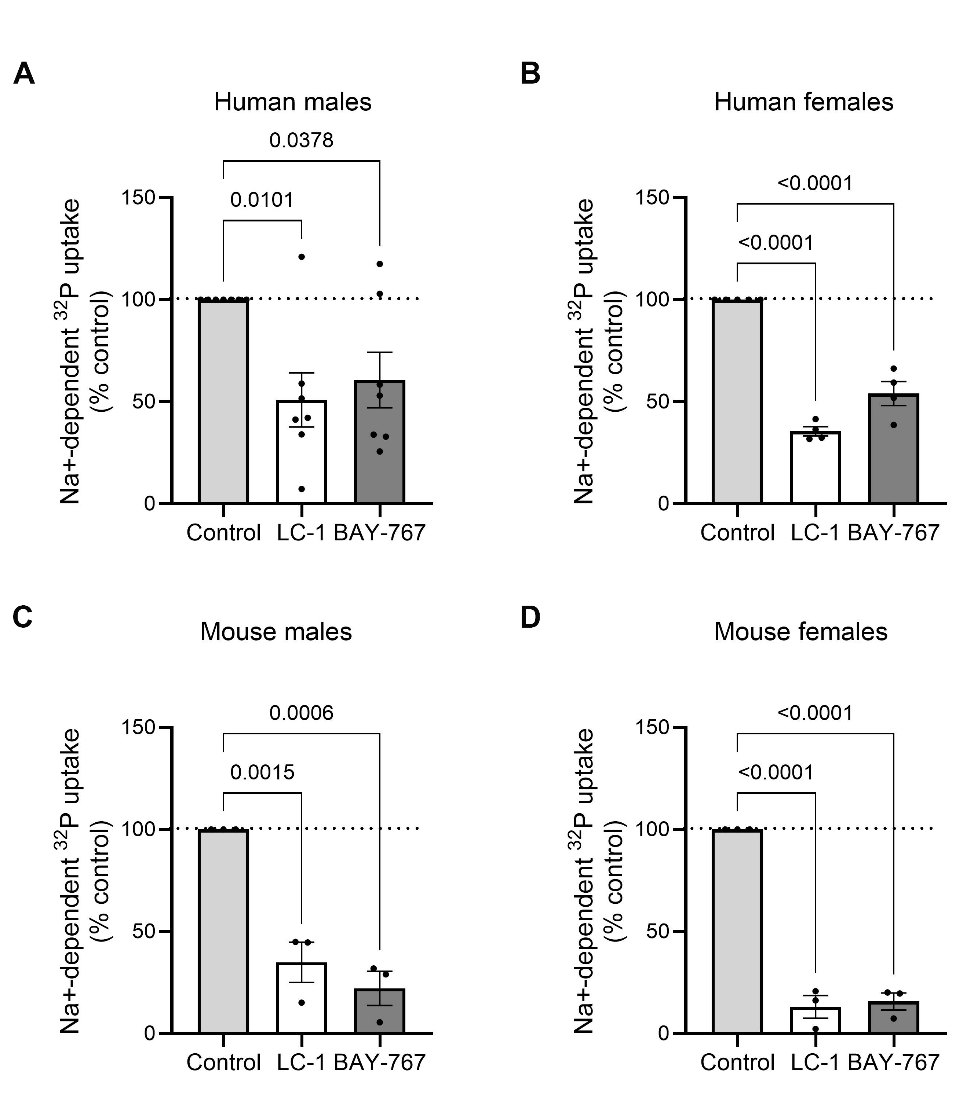
**

**Supplementary Figure S4. Sodium-dependent phosphate uptake into renal BBMVs in the presence of 100μM of the inhibitors LC-1 and BAY-767.** Uptake of phosphate into BBMVs isolated from renal cortices of adult human males (A) and females (B) and from kidneys from 4.5 months old male (C) and female (D) mice. Uptake was determined in the presence of a sodium gradient and in the absence (only 1% DMSO, vehicle present) or presence of 100 μM of either LC-1 or BAY-7676. N = 3-6 samples/group. All bars represent mean ± SEM. Statistical significant differences were analyzed using One-way ANOVA analysis followed by Dunnet's post-hoc test.


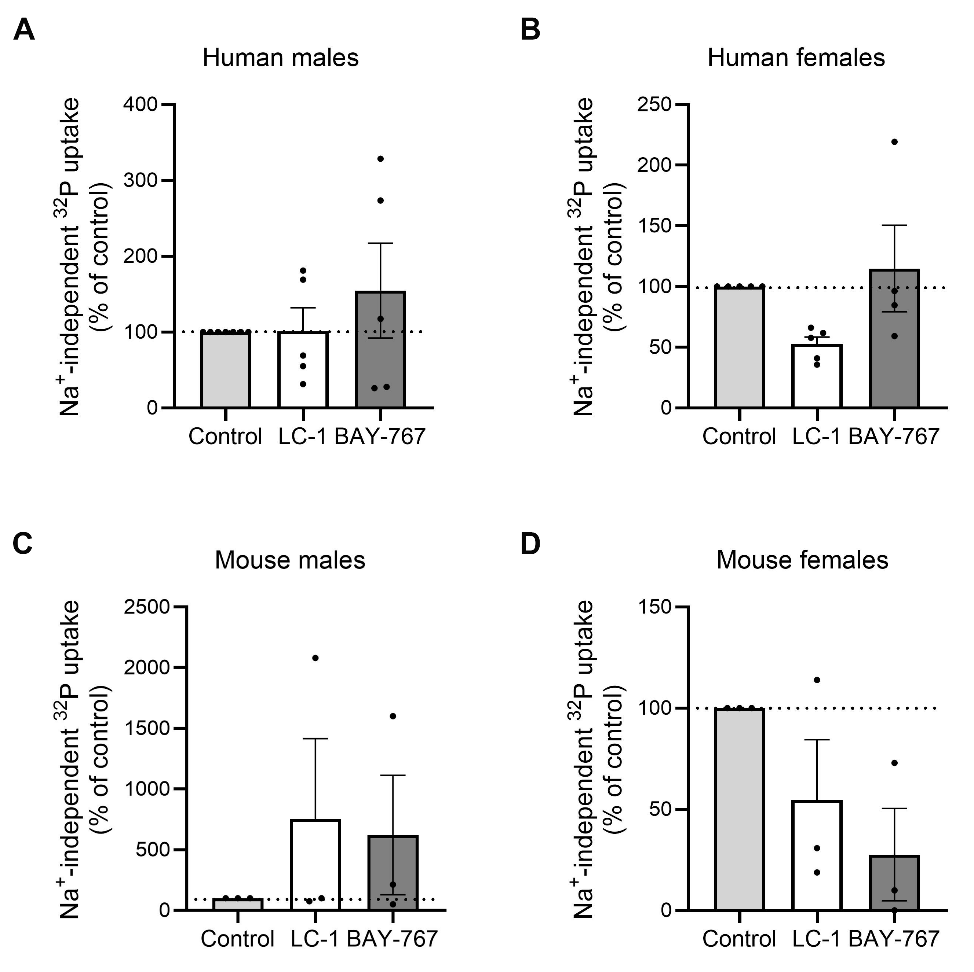


**Supplementary Figure S5. Sodium-independent phosphate uptake into renal BBMVs in the presence of the inhibitors LC-1 and BAY-767.** Uptake of phosphate into BBMVs isolated from renal cortices of adult human males (A) and females (B) and from kidneys from 4.5 months old male (C) and female (D) mice. Uptake was determined in the absence of a sodium gradient and in the absence (1% DMSO, vehicle) or presence of 100 μM of either LC-1 or BAY-7676. N = 3-7 samples/group. All bars represent mean ± SEM. No statistically significant differences were observed using One-way ANOVA analysis.


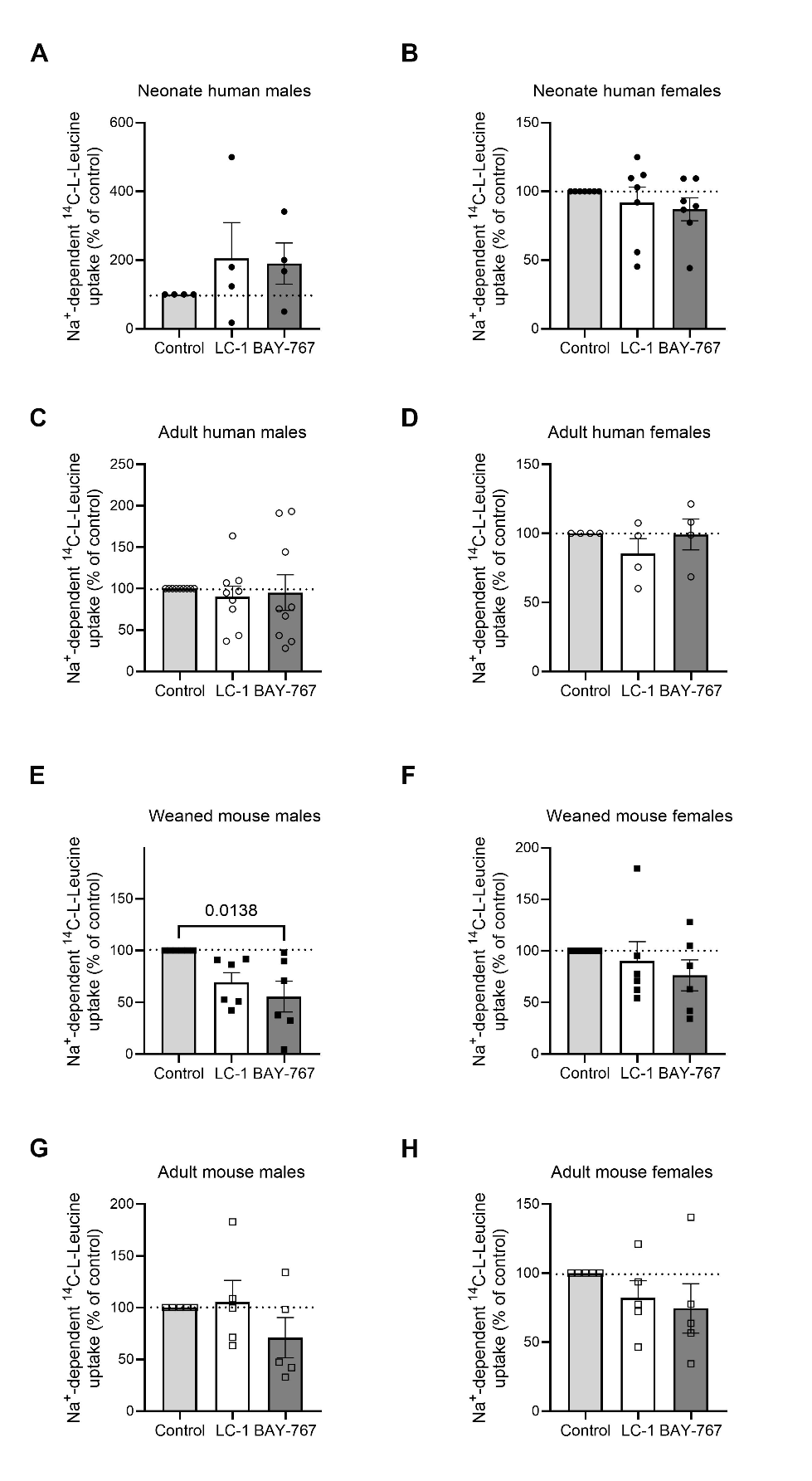


**Supplementary Figure S6. Na^+^-dependent leucine uptake in all experimental groups**. Uptake of ^14^C-leucine into BBMVs isolated from renal cortices from human neonate males (A) and females (B), and human adult males (C) and females (D) as well as from kidneys from weaned male (E) and female (F) C57BL/6J mice and adult male (G) and female (H) C57BL/6N mice. Uptakes were determined in the absence and presence of a sodium gradient and in the absence (1% DMSO, vehicle) or presence of 100 μM of either LC-1 or BAY-7676. N = 4-6 samples/group. All bars represent mean ± SEM. Statistical differences were analyzed using One-way ANOVA analysis followed by Dunnet’s post hoc test.


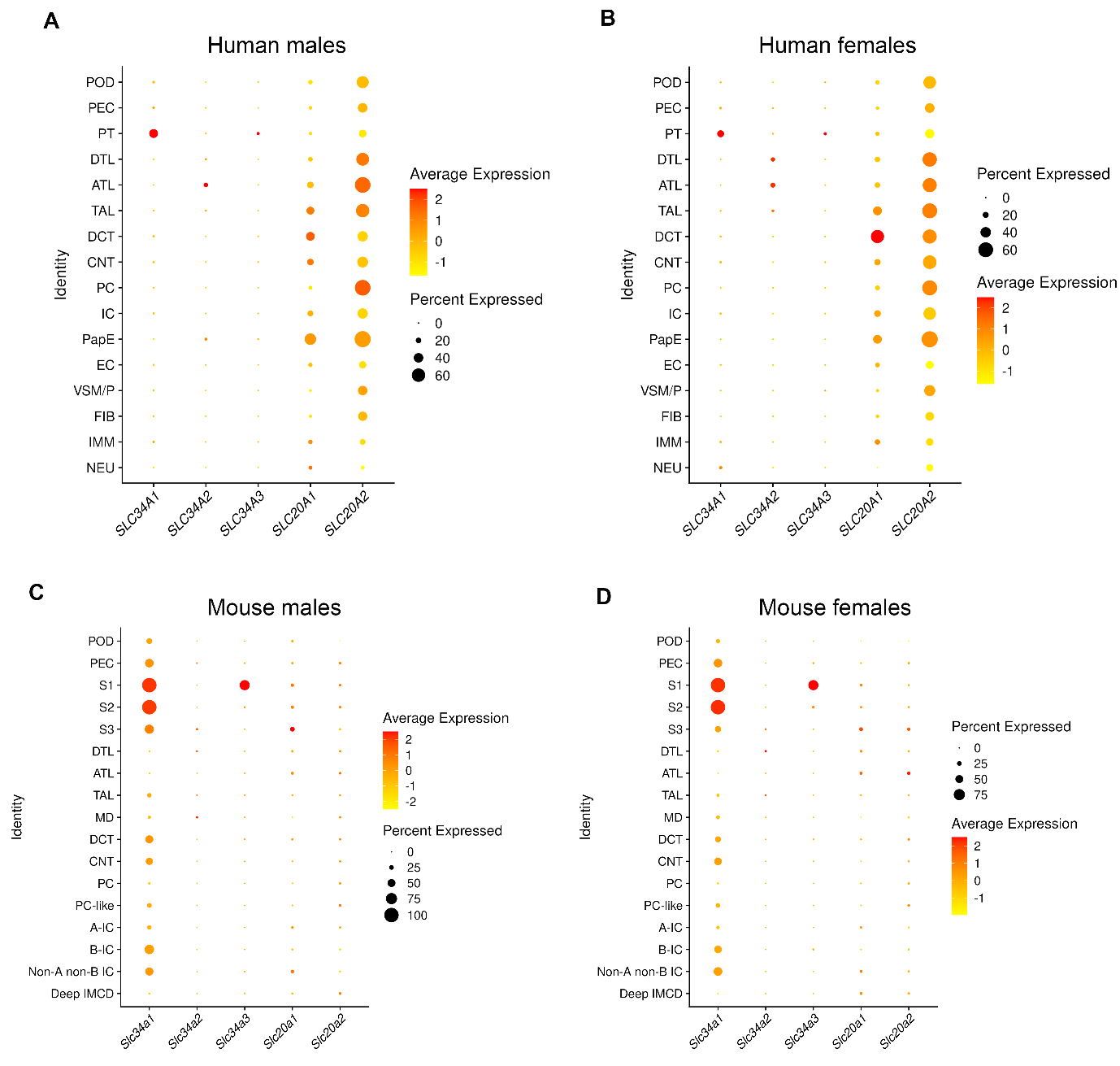


**Supplementary Figure S7. Sex-specific single nuclei and single cell transcriptome data from murine and human kidney.** Expression of *SLC34A1, SLC34A2, SLC34A3, SLC20A1*, and *SLC20A2* in the human single-nuclei ([GSE183279](https://www.ncbi.nlm.nih.gov/geo/query/acc.cgi?acc=GSE183279))^3^ and mouse single-cell ([GSE129798](https://www.ncbi.nlm.nih.gov/geo/query/acc.cgi?acc=GSE129798))^4^ RNA-seq datasets were stratified by sex category. POD = Podocyte, PEC = Parietal Epithelial Cell, PT = Proximal Tubule, S1 = Proximal Tubule Segment 1, S2 = Proximal Tubule Segment 2, S3 = Proximal Tubule Segment 3, DTL = Descending Thin Limb, ATL = Ascending Thin Limb, TAL = Thick Ascending Limb, MD = Macula Densa, DCT = Distal Convoluted Tubule, CNT = Connecting Tubule, PC = Principal Cell, PC-like  =  Principal-like Cell, IC = Intercalated Cell, IC-A  =  Intercalated-A Cell, IC-B = Intercalated-B Cell, IC non-A non-B = Non-A Non-B Intercalated Cell, Deep IMCD = Deep Inner Medullary Collecting Duct, PapE = Papillary Tip Epithelial Cell, EC = Endothelial Cell, VSM/P = Vascular Smooth Muscle Cell/Pericyte, FIB = Fibroblast, IMM = Immune Cell, NEU = Neural Cell.

**
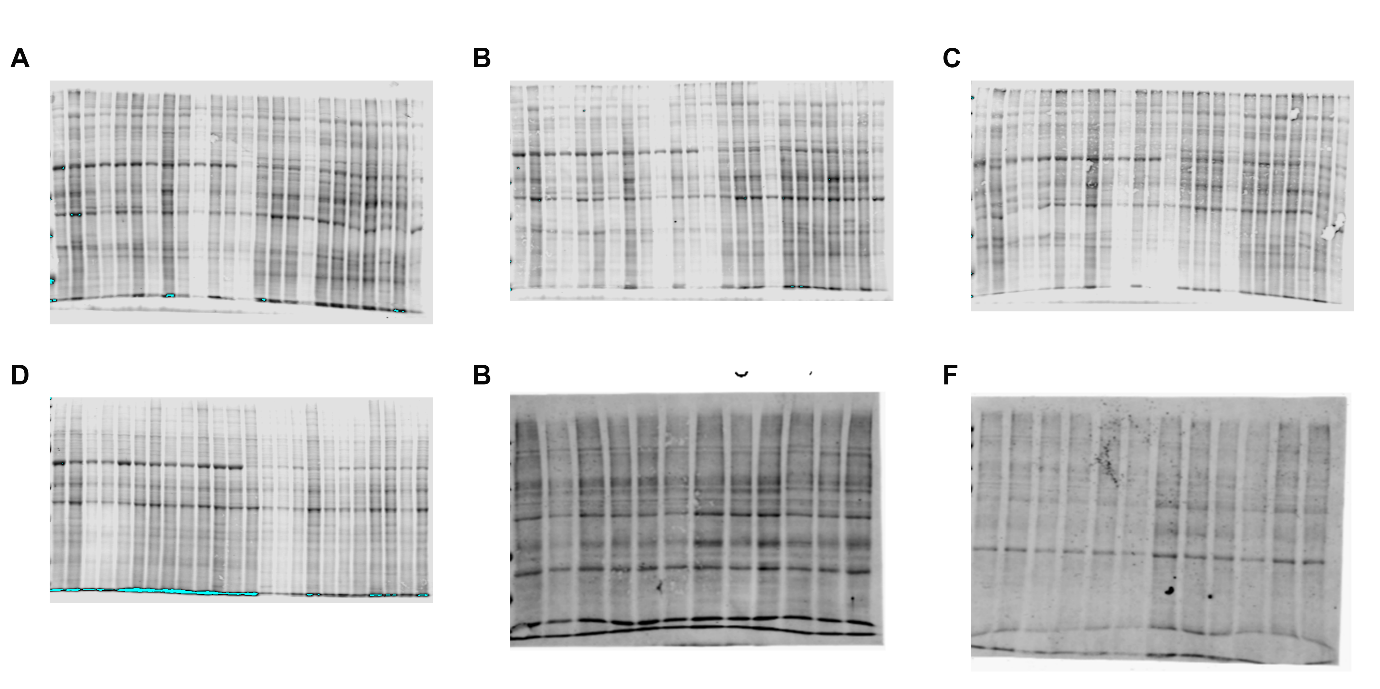
**

**Supplementary Figure S8. Total protein staining.** Total protein staining images from immunoblotting analysis of (A) human NaPi-IIa, (B) human NaPi-IIc, (C) human NaPi-IIb, (D) human Pit1, (E) mouse NaPi-IIa and (F) mouse NaPi-IIc.

**Supplementary Table S1. Renal suitability scores used to determine eligibility of kidneys for study inclusion.** Estimated glomerular filtration rate (eGFR) was calculated using the CKD-EPI 2021 equation ^5^ from hospitalization (initial) to kidney donation (last). For initial and last eGFR, scores were assigned as follows: **0** if eGFR ≥ 60 mL/min/1.73 m²; **1** if eGFR < 60 mL/min/1.73 m². eGFR decline was expressed as a percentage and calculated as *(initial eGFR − last eGFR) / initial eGFR × 100*. Scores for eGFR decline were assigned as: **0** for no decline or initial eGFR ≥ 60 mL/min/1.73 m²; **−1** for initial eGFR < 60 mL/min/1.73 m² and last eGFR ≥ 60 mL/min/1.73 m²; **1** for ≤ 10 % decline; and **2** for > 10 % decline. Fibrosis and kidney injury scores were assessed by densitometric analysis of COL1A1, NGAL, and KIM-1 protein signals, normalized to total protein in Western blot images (Supplementary Figure 1). Scores for fibrosis were assigned as: **0** for COL1A1 densitometry values ≥ 0 and < 1.5; **1** for ≥ 1.5 and ≤ 2 values; and **2** for > 2 values. Scores for kidney injury for NGAL were assigned as: **0** for densitometry values ≥ 0 and < 5; **1** for ≥ 5 and ≤ 10 values; and **2** for > 10 values. Scores for kidney injury for KIM-1 were assigned as: **0** for densitometry values ≥ 0 and < 0.7; **1** for ≥ 0.7 and ≤ 0.8 values; and **2** for > 0.8 values.Total score is the sum of the score for each individual, and classified as suitable (light green, score: 0-2), unsuitable (light red, score > 2). M. Male, F: female.

| **Kidney ID** | **Sex** | **Initial**  **eGFR** | **Last eGFR** | **eGFR decline** | **Fibrosis**  **COL1A1** | **Kidney injury** | | **Total**  **score** |
| --- | --- | --- | --- | --- | --- | --- | --- | --- |
|  |  |  |  |  |  | **NGAL** | **KIM-1** |  |
| K1 | M | 0 | 1 | 2 | 0 | 0 | 0 | 3 |
| K2 | M | 0 | 0 | 0 | 0 | 2 | 0 | 2 |
| K3 | F | 1 | 1 | 2 | 0 | 2 | 1 | 7 |
| K4 | M | 0 | 0 | 0 | 0 | 1 | 0 | 1 |
| K5 | F | 0 | 1 | 0 | 0 | 1 | 0 | 2 |
| K6 | M | 0 | 0 | 0 | 0 | 0 | 0 | 0 |
| K7 | M | 0 | 1 | 2 | 0 | 2 | 0 | 5 |
| K8 | M | 0 | 0 | 0 | 0 | 0 | 0 | 0 |
| K9 | F | 1 | 1 | 0 | 0 | 0 | 0 | 2 |
| K10 | M | 1 | 1 | 0 | 1 | 0 | 1 | 4 |
| K11 | M | 0 | 0 | 0 | 0 | 0 | 2 | 2 |
| K12 | M | 0 | 1 | 2 | 0 | 1 | 0 | 4 |
| K13 | F | 1 | 1 | -1 | 0 | 2 | 2 | 5 |
| K14 | F | 0 | 0 | 0 | 0 | 0 | 0 | 0 |
| K15 | F | 1 | 0 | -1 | 1 | 1 | 0 | 2 |
| K16 | M | 0 | 0 | 0 | 2 | 1 | 0 | 3 |
| K17 | M | 1 | 0 | -1 | 1 | 0 | 1 | 2 |
| K18 | M | 0 | 0 | 0 | 0 | 0 | 0 | 0 |
| K19 | M | 0 | 0 | 0 | 1 | 0 | 0 | 1 |
| K20 | M | 0 | 1 | 2 | 2 | 2 | 0 | 7 |
| K21 | F | 1 | 1 | 2 | 2 | 0 | 0 | 6 |

**Supplementary Table S2. IC_50_ of SLC34 inhibitors in vitro.**

| **Name** | **IC_50_ NaPi-IIa (mM)** | | **IC_50_ NaPi-IIb (mM)** | **IC_50_ NaPi-IIc (mM)** |
| --- | --- | --- | --- | --- |
|  | **Human^a^** | **Mouse^b^** | **Human^a^** | **Human^a^** |
| LC-1 | 73 | 195 | 51 | 1950 |
| BAY-767 | 4.6 | 227 | >50000 | >50000 |

^a^Values were obtained with the membrane potential dye assay in CHO cells stable transfected with human SLC34 proteins or ^b^by electrophysiology after heterologous expression in *Xenopus laevis* oocytes of mouse NaPi-IIa, as described in the method section above.

**Supplementary Table S3.** **Antibodies**

| **Name** | **Working dilution (method^a^)** | **Company** | **Cat. No.** |
| --- | --- | --- | --- |
| Anti-rat-NaPi-IIa  (Nterm Rb-1) | 1:1000 (WB)  1:200 (IF) | Laboratory of Prof. C. A. Wagner, University of Zurich, Switzerland | N/A |
| Anti-human-NaPi-IIb  (644-662 Rb-1) | 1:1000 (WB)  1:200 (IF) | Laboratory of Prof. C. A. Wagner, University of Zurich, Switzerland | N/A |
| Anti-human-NaPi-IIc  (Cterm Rb-2) | 1:1500 (WB)  1:200 (IF) | Laboratory of Prof. C. A. Wagner, University of Zurich, Switzerland | N/A |
| Anti-human-PIT1 | 1:1000 (WB) | Cell Signaling | 12765 |
| Anti-human AQP1 | 1:200 (IF) | Abcam | ab9566 |
| Anti-mouse-NaPi-IIa | 1:1000 (WB) | Laboratory of Prof. H. Murer, University of Zurich, Switzerland ^6^ | N/A |
| Anti-mouse-NaPi-IIc | 1:1000 (WB) | Laboratory of Prof. H. Segawa, Tokushima University, Japan^7^ | N/A |
| Anti-human TIM-1/Kim-1 (E1R9N) rabbit monoclonal IgG | 1:1000 (WB) | Cell Signaling | 14971 |
| Anti-human/mouse/rat Lipocalin-2/ NGAL goat polyclonal IgG | 1:1000 (WB) | R&D Systems | AF1757 |
| Anti-human/mouse/rat COL1A1 (E8F4L) rabbit monoclonal IgG | 1:1000 (WB) | Cell Signaling | 72026 |
| Anti-Rabbit IgG (H+L), HRP conjugate | 1:10000 (WB) | Promega | W401B |
| Anti-Mouse IgG (H+L), HRP conjugate | 1:10000 (WB) | Promega | W402B |
| Donkey anti-Rabbit IgG (H+L) Highly Cross-Adsorbed Secondary Antibody, Alexa Fluor™ 594 | 1:2000 (IF) | Invitrogen | A21207 |
| Donkey anti-Mouse IgG (H+L) Highly Cross-Adsorbed Secondary Antibody, Alexa Fluor™ 488 | 1:2000 (IF) | Invitrogen | A21202 |

^a^Method: Western Blot (WB), immunofluorescence (IF)

**SUPPLEMENTARY REFERENCES**

1. Hernando N, Myakala K, Simona F, et al. Intestinal Depletion of NaPi-IIb/

in Mice: Renal and Hormonal Adaptation. *J Bone Miner Res* 2015; **30**(10): 1925-37.

2. Myakala K, Motta S, Murer H, et al. Renal-specific and inducible depletion of NaPi-IIc/Slc34a3, the cotransporter mutated in HHRH, does not affect phosphate or calcium homeostasis in mice. *Am J Physiol-Renal* 2014; **306**(8): F833-F43.

3. Lake BB, Menon R, Winfree S, et al. An atlas of healthy and injured cell states and niches in the human kidney. *Nature* 2023; **619**(7970): 585-94.

4. Ransick A, Lindstrom NO, Liu J, et al. Single-Cell Profiling Reveals Sex, Lineage, and Regional Diversity in the Mouse Kidney. *Dev Cell* 2019; **51**(3): 399-413 e7.

5. Inker LA, Levey AS. New Equations for Estimating the GFR without Race REPLY. *New Engl J Med* 2022; **386**(17): 1672-3.

6. Custer M, Lotscher M, Biber J, Murer H, Kaissling B. Expression of Na-P-I Cotransport in Rat-Kidney - Localization by Rt-Pcr and Immunohistochemistry. *Am J Physiol* 1994; **266**(5): F767-F74.

7. Segawa H, Kaneko I, Takahashi A, et al. Growth-related renal type II Na/Pi cotransporter. *J Biol Chem* 2002; **277**(22): 19665-72.
